# Activation of a lateral hypothalamic-ventral tegmental circuit gates motivation

**DOI:** 10.1371/journal.pone.0219522

**Published:** 2019-07-10

**Authors:** Felipe L. Schiffino, Justin N. Siemian, Michele Petrella, Brenton T. Laing, Sarah Sarsfield, Cara B. Borja, Anjali Gajendiran, Maria Laura Zuccoli, Yeka Aponte

**Affiliations:** 1 National Institute on Drug Abuse, Intramural Research Program, National Institutes of Health, Baltimore, Maryland, United States of America; 2 Pharmacology Unit, School of Pharmacy, University of Camerino, Camerino (MC), Italy; 3 The Solomon H. Snyder Department of Neuroscience, Johns Hopkins University School of Medicine, Baltimore, Maryland, United States of America; University of Texas at Austin, UNITED STATES

## Abstract

Across species, motivated states such as food-seeking and consumption are essential for survival. The lateral hypothalamus (LH) is known to play a fundamental role in regulating feeding and reward-related behaviors. However, the contributions of neuronal subpopulations in the LH have not been thoroughly identified. Here we examine how lateral hypothalamic leptin receptor-expressing (LH^LEPR^) neurons, a subset of GABAergic cells, regulate motivation in mice. We find that LH^LEPR^ neuronal activation significantly increases progressive ratio (PR) performance, while inhibition decreases responding. Moreover, we mapped LH^LEPR^ axonal projections and demonstrated that they target the ventral tegmental area (VTA), form functional inhibitory synapses with non-dopaminergic VTA neurons, and their activation promotes motivation for food. Finally, we find that LH^LEPR^ neurons also regulate motivation to obtain water, suggesting that they may play a generalized role in motivation. Together, these results identify LH^LEPR^ neurons as modulators within a hypothalamic-ventral tegmental circuit that gates motivation.

## Introduction

The hypothalamus exerts control over homeostatic functions and behavioral states critical to survival. While much of the hypothalamus can be divided by gene expression [1–3], function [4], and classical neuroanatomical boundaries [5], the lateral hypothalamic area contains genetically heterogeneous neuronal populations whose function and connectivity have only partially been characterized. A fundamental role of the lateral hypothalamus (LH) in regulating appetitive and reward-related behaviors has been evident for decades [6], but the contributions of specific genetically-identified neuronal types to such phenomena have not been unraveled.

Intermingled within the LH are neurons defined by neurotransmitter markers such as vesicular GABA transporter (SLC32A1 commonly known as VGAT) [6–8] and vesicular glutamate transporter 2 (SLC17A6 commonly known as VGLUT2) [6, 8, 9]. Moreover, many of these neurons can be identified by expression of neuropeptides, including hypocretin (orexin; HCRT), melanin-concentrating hormone (MCH), neurotensin (NTS), and galanin (GAL) as well as receptors such as leptin receptor (LEPR) [10–16]. Due to this heterogeneity, it is not surprising that LH electrical stimulation evokes a variety of behavioral effects including feeding, grooming, gnawing, sexual behavior, motivation, and reinforcement [17–19]. Recent studies using optogenetics and chemogenetics further support this idea by showing that activation of LH GABAergic neurons promotes feeding and reward-seeking behaviors [20], whereas activation of LH glutamatergic neurons suppresses feeding and is aversive [8]. Moreover, optogenetic activation of LH GABAergic, but not glutamatergic, projections in the ventral tegmental area (VTA) increases food intake in sated mice [21] as well as other motivated behaviors such as social interaction [22]. Furthermore, the observation that lateral hypothalamic stimulation is reinforcing in some conditions but evokes a hunger-like state in others is paradoxical [23], but may suggest that the rewarding effects of stimulation are governed by circuits within the LH and their projections that are distinct from those regulating feeding-related behaviors. In support of this, *in vivo* calcium imaging experiments have begun to reveal that distinct, predominantly non-overlapping populations of LH GABAergic neurons become active during appetitive and consummatory behaviors [20]. Since lateral hypothalamic GABAergic and glutamatergic neurons can be further divided into several subpopulations defined by the expression of neuropeptides and receptors [24], it is likely that specific genetically-distinct cell types within the LH differentially encode for either feeding- or reward-related behaviors.

Leptin receptor-expressing neurons are a distinct subpopulation of GABAergic neurons in the LH (LH^LEPR^) [13]. These LH^LEPR^ neurons send axonal projections to several brain regions, including the VTA, and appear to regulate mesolimbic dopamine production [13], suggesting a potential role in reward processing and motivated behavior. However, studies have not yet demonstrated that LH^LEPR^ neurons form functional synapses with VTA neurons. Therefore, the role of LH^LEPR^ neurons in regulating motivated behaviors and the potential downstream circuits in which they participate remain unclear.

To investigate this, we trained mice to obtain food pellets on a progressive ratio (PR) schedule of reinforcement [25] and assessed the effects of manipulation of LH^LEPR^ neuronal activity on motivated behaviors. We first examined the effects of chemogenetic activation or inhibition of LH^LEPR^ neurons on PR performance, followed by a combination of optogenetic and electrophysiological assays to determine whether LH^LEPR^ neurons are synaptically connected to cells within the VTA. We then optogenetically stimulated LH^LEPR^ axonal projections in the VTA to determine whether this pathway regulates PR performance. Finally, we investigated whether LH^LEPR^ neurons promote motivation specific for food rather than other drives by measuring the effects of optogenetic manipulation of LH^LEPR^ neuronal activity on motivation to obtain water.

## Materials and methods

### Animals

Two- to four-month-old male and female *Lepr*^*Cre/+*^ (*Lepr*^*tm3(Cre)Mgmj*^; C57BL/6J background; kindly provided by M.G. Myers Jr., University of Michigan Medical School, MI, USA) [26] mice and *Lepr*^*Cre/+*^*;Rosa26*^*YFP/YFP*^ mice (*Lepr*^*Cre*^ crossed to *Gt(ROSA)26Sor*^*tm3(CAG-EYFP)Hze*^; C57BL/6J background; Strain 7903, The Jackson Laboratory, ME, USA) were used in this study. Prior to stereotaxic viral injection, mice were group housed with littermates in temperature- and humidity-controlled rooms with *ad libitum* access to water and rodent chow (PicoLab Rodent Diet 20, 5053 tablet, LabDiet/Land O’Lakes Inc., MO, USA) on a 12 h light/dark cycle.

### Study approval

All experimental protocols were conducted in accordance with U.S. National Institutes of Health Guidelines for the Care and Use of Laboratory Animals and with the approval of the National Institute on Drug Abuse Animal Care and Use Committee (protocols 16-CNRB-116 and 16-CNRB-126). All surgeries were performed under isoflurane anesthesia, and all efforts were made to minimize suffering.

### Stereotaxic viral injection

For behavioral experiments using chemogenetic techniques, six to eight-week-old *Lepr*^*Cre*^ mice were used. Mice were anesthetized with isoflurane and placed onto a stereotaxic apparatus (David Kopf Instruments, CA, USA). After exposing the skull by a minor incision, small holes (< 1 mm diameter) were drilled bilaterally for virus injection. An adeno-associated virus (rAAV2/rh10-hSyn-DIO-hM3D(Gq):mCherry or rAAV2/rh10-hSyn-DIO-hM4D(Gi):mCherry; titer: 3×10^12^ virus molecules/ml each virus; University of North Carolina Vector Core, NC, USA; [27]) was injected bilaterally (30 nl; rate: 30 nl/min) into the lateral hypothalamus (LH; bregma, –1.55 mm; midline, ±1.10 mm; dorsal surface, −5.20 mm) by a pulled glass pipette (20–30 μm inner diameter) with a micromanipulator (Narishige International USA Inc., NY, USA) controlling the injection speed. Subsequently, the incision was stitched, and mice were individually housed for 2–5 weeks for post-surgical recovery and viral transduction.

For mapping LH^LEPR^ axonal projections, eight-week-old *Lepr*^*Cre*^ mice were unilaterally injected with 2.5–5 nl of rAAV2/9-CAG-FLEX-eGFP-WPRE-bGH or 30 nl of rAAV2/9-hEF1α-DIO-synaptophysin-mCherry (titer: 2.28×10^13^ genomic copies/ml and 1.0×10^13^ genomic copies/ml, respectively; University of Pennsylvania Gene Therapy Program Vector Core, PA, USA and Massachusetts Institute of Technology Viral Gene Transfer Core, MA, USA, respectively; [28]) into the LH as described above. After surgery, mice were individually housed for 6 weeks for post-surgical recovery and viral transduction.

For behavioral experiments using optogenetic techniques to manipulate LH^LEPR^ neuronal activity, six- to eight-week-old *Lepr*^*Cre*^ mice were bilaterally injected with 40 nl of an adeno-associated virus (rAAV2/9-CAG-FLEX-*rev*-ChR2:tdTomato, titer: 4.87×10^12^ genomic copies/ml; rAAV2/9-CAG-FLEX-ArchT:GFP, titer: 4.70×10^12^ genomic copies/ml; or rAAV2/9-CAG-FLEX-GFP, titer: 2.30×10^13^ genomic copies/ml; University of Pennsylvania Gene Therapy Program Vector Core, PA, USA and University of North Carolina Vector Core, NC, USA; [29, 30]) into the LH (bregma, –1.55 mm; midline, ±1.10 mm; skull surface, –5.20 mm) as described above. Optical fibers were implanted bilaterally at 10° angles above LH^LEPR^ somas (bregma, –1.55 mm; midline, ±1.70 mm; dorsal surface, –4.50 mm). For behavioral experiments using optogenetic techniques to manipulate LH^LEPR^ axonal projections in the VTA, six to eight-week-old *Lepr*^*Cre*^ mice were bilaterally injected with 50 nl of an adeno-associated virus (rAAV2/rh10-CAG-FLEX-*rev*-ChR2:tdTomato, titer: 8.43×10^12^ genomic copies/ml or rAAV2/9-CAG-FLEX-GFP, titer: 2.30×10^13^ genomic copies/ml; University of Pennsylvania Gene Therapy Program Vector Core, PA, USA) into the LH (bregma, –1.55 mm; midline, ±1.10 mm; skull surface, –5.20 mm) as described above. Optical fibers were implanted bilaterally at 10° angles above LH^LEPR^ fibers in the ventral tegmental area (VTA; bregma, –3.00 mm; midline, ±2.20 mm; skull surface, –4.20 mm). Fiber implants were affixed to the skull with cyanoacrylate adhesive and C&B Metabond Quick Adhesive Cement System (Parkell, Inc., NY, USA). Subsequently, mice were individually housed for 2–5 weeks for post-surgical recovery and viral transduction.

For brain slice electrophysiological recordings, two to four-month-old *Lepr*^*Cre*^ mice were bilaterally injected with 30–50 nl of an adeno-associated virus (rAAV2/rh10-CAG-FLEX-*rev*-ChR2:tdTomato) into the LH as described above. Recordings were performed 2–5 weeks after post-surgical recovery and viral transduction.

### Behavioral experiments

During the first week of post-surgical recovery all mice were individually housed and provided with *ad libitum* access to water and rodent chow. During the second week and for all food experiments, mice were weighed and food-restricted as follows. Mice were fed approximately 2–3 g standard chow to maintain them at 90% of their *ad libitum* weight. To acclimate to the reinforcer (20 mg food pellets of identical composition to the standard chow), prior to daily rations of standard chow, mice were given 10 pellets per day in their home cages for three days prior to training. For the procedure, mice were transferred to experimental cages with two retractable levers (Coulbourn Instruments LLC, PA, USA). Mice were subjected to one session per day during the light phase. During the first session, mice were trained to retrieve pellets from the food cup through delivery of 30 pellets dispensed at random intervals over the course of a 60 min period. Over the next two sessions, mice were trained to press the active lever to receive a single pellet per press (fixed ratio; FR1), while presses of the inactive lever had no programmed consequence. FR1 sessions lasted for 60 min or until 40 pellets were earned, whichever occurred first. For the next two sessions (60 min each) the active lever press requirement was increased to three lever presses for one pellet (FR3). Then, for twelve sessions (120 min each), animals were trained on a progressive ratio schedule (PR) according to the following formula: Response Requirement = (5*e* ^0.2 × Pellet Number^)– 5, as previously described [25]. Importantly, after the sixth PR session, mice used in experiments for chemogenetic activation or photostimulation (*i*.*e*. LH^LEPR/hM3D^, LH^LEPR/ChR2^, and LH^LEPR/ChR2^ → VTA) were given *ad libitum* access to food in their home cages for the remainder of the procedure. Mice used in chemogenetic inhibition experiments (*i*.*e*. LH^LEPR/hM4D^) were maintained on food restriction as described above. For water experiments, identical training was used with the exception that 0.25 ml of water was delivered via a liquid solenoid valve (Coulbourn Instruments LLC, PA, USA) instead of food pellets. All mice were water-restricted (1 min access per day) during initial training. Then, after learning the task, LH^LEPR/ChR2^ mice had *ad libitum* access to water in their home cages throughout the course of testing, while LH^LEPR/ArchT^ mice remained water-restricted. When bidirectional effects of chemogenetic or optogenetic manipulations were assessed, the same group of control mice was used as reference for both the restricted and sated condition. LH^LEPR/mCherry^ or LH^LEPR/GFP^ control groups were first trained and tested identically to LH^LEPR/hM4D^ or LH^LEPR/ArchT^ mice under food- or water-restricted conditions, respectively, and were subsequently returned to *ad libitum* access and tested again under food- or water-sated conditions.

For chemogenetic activation and inhibition experiments, mice were injected intraperitoneally with clozapine *N*-oxide (CNO; 1 mg/kg, 2 mg/kg, or 4 mg/kg) or sterile water (vehicle) 20 min prior to each PR test session. Drug dose and vehicle administration were counterbalanced between mice, and at least 48 h separated each PR test session.

For optogenetic manipulation of LH^LEPR^ neurons during PR experiments, mice were photostimulated with 10-ms 450 nm laser light pulses at a frequency of 20 Hz for 1 min. The stimulus was applied every 10 minutes for 2 h. For photoinhibition, constant laser light (1 s; 520 nm) was delivered every other second for 2 h. For photostimulation of LH^LEPR/ChR2^ →VTA, 10-ms 450 nm laser light pulses at a frequency of 10 or 20 Hz for 1 min were applied.

For chemogenetic locomotion experiments, mice were injected with 1 mg/kg CNO. 20 min later, mice were placed in open field chambers (dimensions: 30 cm × 27 cm × 30 cm) equipped with ANY-maze animal tracking systems (Stoelting Co., IL, USA) for 2 hr, and the total distance traveled was calculated. For optogenetic locomotor experiments, patch cords were attached, and mice were placed into open field chambers for 48 min. Alternating 3-min epochs were paired with photostimulation for 8 blocks each of ON-OFF or OFF-ON; the order was counterbalanced across mice. The total distance traveled in the ON epochs or the OFF epochs was calculated.

### Slice preparation and electrophysiology

For voltage clamp recordings, mice were deeply anesthetized with isoflurane, and after decapitation, brains were rapidly removed and placed into an ice-cold N-methyl-D-glucamine (NMDG)-based slicing solution [31] containing (in mM): 92 NMDG, 20 HEPES, 25 glucose, 30 NaHCO_3_, 1.2 NaH_2_PO_4_, 2.5 KCl, 5 sodium ascorbate, 3 sodium pyruvate, 2 thiourea, 10 MgSO_4_, and 0.5 CaCl_2_, pH 7.4, and osmolarity of 307–314 mOsm. For current clamp recordings, deeply anesthetized mice were intracardially perfused with room-temperature NMDG-based slicing solution and were then decapitated and brains were rapidly removed into a room-temperature NMDG solution. Acute horizontal brain slices (180 μm thick) containing the ventral tegmental area (VTA) or coronal brain slices (200 μm) were obtained using a vibratome (Leica VT1200S, Leica Biosystems Inc., IL, USA). Brain slices were transferred to a holding chamber filled with a solution containing (in mM): 92 NaCl, 20 HEPES, 25 glucose, 30 NaHCO_3_, 1.2 NaH_2_PO_4_, 2.5 KCl, 5 sodium ascorbate, 3 sodium pyruvate, 2 thiourea, 1 MgSO_4_, and 2 CaCl_2_ (pH 7.4, 307–310 mOsm). For all electrophysiological recordings, a single slice was transferred to the recording chamber and continuously perfused at a flow rate of 1.5 to 2.0 ml/min with artificial cerebrospinal fluid (aCSF, in mM): 125 NaCl, 2.5 KCl, 1.25 NaH_2_PO_4_, 1 MgCl_2_.6H_2_O, 11 glucose, 26 NaHCO_3_, 2.4 CaCl_2_, pH 7.4, and osmolarity of 307–310 mOsm). All solutions were saturated with 95% O_2_ and 5% CO_2_. Voltage clamp recordings were conducted at 32°C while current clamp recordings were conducted at room temperature.

For channelrhodopsin (ChR2)-assisted circuit mapping (CRACM) of neurons in the VTA synaptically connected to LH^LEPR^ neurons, *Lepr*^*Cre*^ mice were bilaterally injected with an adeno-associated virus into the LH as previously described. Horizontal slices containing the VTA from AAV-injected *Lepr*^*Cre*^ mice were used and ChR2:tdTomato-containing axons visualized in the VTA. The VTA was identified as being medial to the medial terminal nucleus of the accessory optic tract (MT), and the recordings were performed in the lateral part of the VTA. Neurons were visualized first with epifluorescence, followed by infrared differential interference contrast (IR-DIC) optics on an Olympus BX51WI microscope (Olympus Corporation, MA, USA). Whole-cell voltage clamp recordings of VTA neurons were performed using patch pipettes (2.5–5.0 MΩ) containing (in mM): 117 cesium methanesulfonate, 20 HEPES, 0.4 EGTA, 2.8 NaCl, 5 TEA-Cl, 4 Mg-ATP, 0.4 Na-GTP, 3 QX-314, and 0.2% biocytin (pH adjusted to 7.3 using CsOH, and osmolality of 287 mOsm). Voltage clamp recordings were performed using an Axopatch 200B amplifier (2 kHz low-pass Bessel filter and 10 kHz digitization using a NI BNC-2090A, National Instruments Corporation, TX, USA) with WinLTP software 2.20b (WinLTP Ltd, Bristol, UK). Recorded neurons were held at 0 mV and photocurrents were evoked by 5-ms blue (473 nm) light pulses (diode-pumped solid-state laser; OptoEngine LLC, UT, USA) delivered at a frequency of 0.1 Hz. For voltage clamp recordings, series resistance (15–30 MΩ) was monitored with a –10 mV hyperpolarizing pulse given every 10 s, and only recordings that remained stable over the period of data collection were used. Light-evoked GABAergic currents were blocked by perfusing the GABA_A_ receptor antagonist picrotoxin (100 μM). Peak current amplitude was measured with Clampfit v10.6 (Molecular Devices LLC, CA, USA) using the average of 15 photostimulation sweeps.

Whole-cell current clamp recordings were conducted using patch pipettes (2.5–5.0 MΩ) containing (in mM): 135 potassium gluconate, 10 HEPES, 4 KCl, 4 MgATP, 0.3 Na_3_GTP (pH adjusted to 7.3 using KOH, 279–285 mOsm). For current clamp recordings, after seal stabilization, we conducted a 2−3-minute baseline period in aCSF followed by perfusion of CNO (5 μM), which was then washed out with normal aCSF. Next, using the same cells in a separate set of recordings we applied tetrodotoxin (1 μM) for 2−3 minutes followed by CNO perfusion to test for action potential-independent depolarization. Solution transit time for current clamp recordings was approximately one minute. All chemicals were obtained from Sigma-Aldrich (MO, USA) or Tocris Bioscience (Bristol, UK). Current clamp analysis was conducted using Clampfit v10.6.

### Immunohistochemistry for electrophysiology

Following voltage clamp electrophysiological recordings, slices were post-fixed in 4% PFA in 1× PBS for 2 h at room temperature. Slices were then incubated in a solution of Avidin-488 (1:500, Vector Labs, CA, USA) for 2 h in 1× PBS. Following 4 × 10 min PBS washes, slices were blocked with 1× PBS/0.3% Triton X-100 with 10% normal donkey serum (NDS) for 1 h at room temperature. Slices were then incubated with chicken anti-TH antibody (1:1000, Aves Labs, Inc., OR, USA) in a solution of 1× PBS/0.3% Triton X-100/2% NDS for 14–18 h at 4°C. Slices were washed in 1× PBS (4 × 10 min each) and then incubated for 3 h with goat anti-chicken AlexaFluor 647 (1:500, Invitrogen, CA, USA) in 1× PBS/0.3% Triton X-100/2% NDS at room temperature. Following PBS washes (4 × 10 min each), slices were mounted onto Superfrost Plus glass slides (VWR International, PA, USA) and coverslipped with DAPI-Fluoromount-G aqueous mounting medium (Electron Microscopy Sciences, PA, USA).

### Immunohistochemistry

Mice were deeply anesthetized with isoflurane and transcardially perfused with 1× PBS followed by 4% PFA in 1× PBS. Whole brains were removed and post-fixed in 4% PFA/PBS overnight at 4°C and subsequently transferred to 1× PBS for storage at 4°C until further processing. Coronal brain sections (50 μm thick) were collected in 1× PBS using a vibratome (Leica VT1200). Freely floating slices were washed with 1× PBS (6 × 10 min) and then incubated with a blocking solution of 1× PBS/0.3% Triton X-100 (PBT) plus 3% normal donkey serum (NDS) for 1 h at room temperature. Sections were then incubated with rabbit anti-DsRed antibody (1:500; Takara Bio USA, Inc., CA, USA), rabbit anti-GFP antibody (1:500; Invitrogen, CA, USA), goat anti-FOS antibody (1:500; Santa Cruz Biotechnology, Inc., TX, USA), or goat anti-PMCH antibody (1:500; Santa Cruz Biotechnology, Inc.) and rabbit anti-HCRT antibody (1:5000; Phoenix Pharmaceuticals, Inc., CA, USA) in 1× PBS/0.3% Triton X-100/3% NDS for 14–18 h at room temperature. After rinsing 6 × 10 min in 1× PBS, sections were incubated for 1 h at room temperature with donkey anti-rabbit-Alexa Fluor 488 (1:500; Invitrogen, CA, USA; for anti-DsRed or anti-HCRT immunofluorescence), donkey anti-rabbit AlexaFluor 594 (1:500; Invitrogen; for anti-GFP immunofluorescence), or goat anti-donkey-Alexa Fluor 647 (1:500; Invitrogen; for anti-PMCH or anti-FOS immunofluorescence) in 1× PBS/0.3% Triton X-100/2% NDS and then washed with 1× PBS (3 × 10 min). Sections were mounted with DAPI-Fluoromount-G aqueous mounting medium (Electron Microscopy Sciences, PA, USA) onto Superfrost Plus glass slides (VWR International, PA, USA).

For phosphorylated STAT3 (pSTAT3) immunofluorescence, sated mice were pretreated with leptin (5 mg/kg i.p.) or saline and euthanized 2 h post-injection. Following collection of brain tissue as described above, sections were washed in 1× PBS (6 × 10 min) and then incubated with the following solutions (prepared in water): 1% NaOH/1% H_2_O_2_ for 20 min; 0.6% glycine for 10 min; 0.03% SDS for 10 min. Then, slices were placed into blocking solution as described above for 1 h. After blocking, sections were incubated overnight in anti-pSTAT3 antibody in blocking solution (1:1000; Cell Signaling Technology, MA, USA). The following day, slices were washed in 1× PBS (6 × 10 min) and incubated with donkey anti-rabbit Alexa Fluor 594 secondary antibody (1:500; Invitrogen) as described above. Sections were mounted with DAPI-Fluoromount-G aqueous mounting medium (Electron Microscopy Sciences) onto Superfrost Plus glass slides (VWR International).

Images were taken with an AxioZoom.V16 fluorescence microscope and tiled z-stacks were collected using an LSM700 laser scanning confocal microscope (Carl Zeiss Microscopy LLC). Images for FOS colocalization experiments were acquired using an LSM700 laser scanning confocal microscope with identical imaging parameters for acquisition of all images. For behavior experiments, mice with mistargeted viral injections or optical fiber placement were excluded from behavioral analyses.

### Fluorescence *in situ* hybridization

Wildtype mice were deeply anesthetized with isoflurane followed by cervical dislocation. Brains were dissected and rapidly frozen in –80°C isopentane, then subsequently stored at –80°C. Coronal cryosections (16 μm) containing the lateral hypothalamus were sliced using a Leica CM3050 S cryostat (Leica Biosystems Inc.) and sections were collected onto Superfrost Plus glass slides (VWR International). Slides were stored at –80°C prior to processing. Fluorescent *in situ* hybridization was performed using the RNAscope Multiplex Fluorescent Assay for fresh frozen tissue (Advanced Cell Diagnostics Inc., CA, USA). Briefly, sections were fixed in 4% PFA in PBS, dehydrated by ethanol series, and treated with Protease IV. Sections were incubated with target probes for mouse leptin receptor (*Lepr*, accession number U42467.1, target region aa1361-2317), neurotensin (*Nts*, accession number NM_024435.2, target region aa2-1188), and vesicular GABA transporter (*Slc32a1 (Vgat)*, accession number NM_009508.2, target region aa894-2037). After hybridization, a series of signal amplification steps (Amp1, Amp2, and Amp3) were performed per kit protocol followed by incubation with labels (Amp4A) for fluorescent visualization of each probe: *Lepr* (Alexa488), *Nts* (Atto550), and *Vgat* (Atto647). Slides were counterstained with DAPI and coverslipped with Fluoromount-G aqueous mounting medium (Electron Microscopy Systems). Tiled 10 μm z-stack images were obtained using an LSM700 confocal microscope (Carl Zeiss Microscopy). LH^LEPR^ neurons were manually assessed for co-expression of *Lepr* with *Nts* and *Vgat*. Cell counts were performed on every fourth brain slice restricted to the region above the fornix that was targeted for behavioral testing; 700 μm x 700 μm grids were positioned bilaterally in the lateral hypothalamus spanning –1.3 to –1.7 mm from bregma such that the bottom center of each grid touched the ventral most surface of the fornix. DAPI-positive cells within and touching the boundaries of the grid were counted. For each probe, a cell was counted positive if four or more fluorescent puncta were associated with a DAPI-stained nucleus.

### Statistics

Behavioral data were collected with GraphicState v4 software (Coulbourn Instruments, Inc.). All data are plotted as mean ± s.e.m., and individual data points are shown for behavior experiments. Statistical analyses were performed using GraphPad Prism v7.0 (GraphPad Software, CA, USA) and OriginPro v9.2 (OriginLab Corporation, MA, USA). Statistical significance for food pellets earned, inactive lever presses, and locomotion were determined by two-way mixed model ANOVA, followed by Bonferroni’s post-test correction for multiple comparisons. Wilcoxon matched-pairs signed rank test was used to analyze active lever final ratio data due to the non-parametric nature of the progressive ratio. In some cases, repeated-measures one-way ANOVA was used to assess dose- or frequency-dependence, followed by Bonferroni’s post-test corrections for multiple comparisons to determine significance from baseline. Two-tailed Student’s *t* tests and repeated-measures ANOVA with Tukey’s post-test for multiple comparisons were used for cell count and electrophysiology experiments. No statistical methods were used to determine sample sizes *a priori*, but sample sizes used are similar to those in other publications utilizing similar methodologies [32, 33]. Experimenters were not blinded to groups, but behavioral data were collected via computer software to minimize bias. Data from electrophysiological recordings were analyzed with Clampfit v10.6 (Molecular Devices LLC, CA, USA), Origin Pro v9.2 (OriginLab Corporation, MA, USA), and MATLAB R2015A (The MathWorks Inc., MA, USA). A *p* value of 0.05 or lower was defined as significant for all experiments.

## Results

### Progressive ratio performance is strengthened by activation of LH^LEPR^ neurons

**Fig 1:**
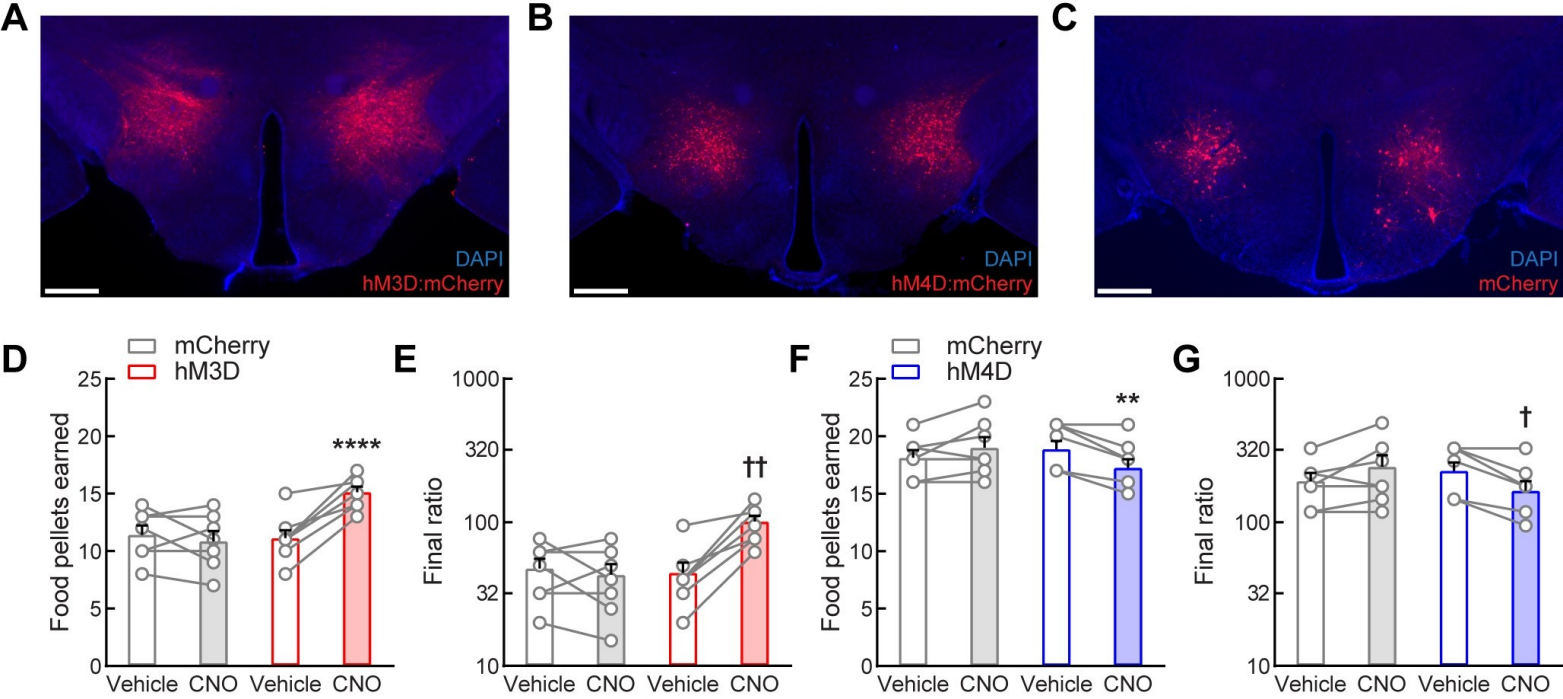
LH^LEPR^ neuronal activity regulates motivation for food reward. (**A−C**) Representative images showing bilateral (**A**) hM3D:mCherry, (**B**) hM4D:mCherry, or (**C**) mCherry expression (red) in LH^LEPR^ neurons. Scale bars: 500 μm. Sections were counterstained with DAPI (blue). Chemogenetic activation of LH^LEPR^ neurons by i.p. injections of CNO (1 mg/kg body weight) significantly increased the number of (**D**) food pellets earned and (**E**) final ratio completed in mice responding on a PR schedule of food delivery during a two-hour session. Chemogenetic inhibition significantly decreased the number of (**F**) food pellets earned and (**G**) final ratio completed. Bars represent mean ± s.e.m.; circles indicate data from individual mice. *n* = 8 mice per group for hM3D and hM4D, *n* = 7 mice for mCherry. Two-way ANOVA with Bonferroni post-test for pellets earned, **p < 0.01, ****p < 0.0001. Non-parametric Wilcoxon matched-pairs signed rank test for final ratio completed, ^†^*p* < 0.05, ^††^*p* < 0.01.

We first investigated whether LH^LEPR^ neurons regulate the performance of mice that have been trained under a PR schedule of responding for food pellets. We used chemogenetic approaches to acutely activate and inhibit these neurons during the PR task. We targeted these cells in the LH of *Lepr*^*Cre*^ mice [26] by bilaterally injecting a Cre recombinase-dependent viral vector that drives the expression of the fluorophore mCherry (control; LH^LEPR/mCherry^) or the excitatory (hM3D; LH^LEPR/hM3D^) or inhibitory (hM4D; LH^LEPR/hM4D^) G-protein-coupled receptor fused to mCherry [34] (Fig 1A–1C and S1A and S1B Fig). Subsequently, mice were trained to lever press for food pellets on a progressive ratio schedule of reinforcement (Fig 1D–1G and S1C–S1G Fig). During this training, all mice were initially food-restricted to 90% of baseline body weight to increase their drive to learn and perform the task. However, after learning the task, LH^LEPR/hM3D^ mice were returned to *ad libitum* food access in their home cages for the duration of the study, while LH^LEPR/hM4D^ mice remained food-restricted. These differences in feeding conditions resulted in an increased performance of the food-restricted mice on the PR task when compared to the fed mice, which was by design to avoid floor and ceiling effects during testing [27]. We next activated LH^LEPR/hM3D^ neurons by intraperitoneal (i.p.) injection of clozapine *N*-oxide (CNO) 20 min prior to the PR test. We observed that chemogenetic activation of LH^LEPR/hM3D^ neurons significantly increased the number of food pellets earned compared to vehicle control and that CNO injection did not affect PR responding for food pellets in the LH^LEPR/mCherry^ control mice (two-way mixed-model ANOVA group × CNO interaction, *F*(1, 13) = 24.31, *p* = 0.0003; Bonferroni post-test, ****p < 0.0001; Fig 1D). Further analysis revealed that activation of LH^LEPR/hM3D^ neurons significantly increased the final ratio completed compared to vehicle control (Wilcoxon matched-pairs signed rank test, ††*p* = 0.0078) and no changes were observed in the LH^LEPR/mCherry^ control mice after CNO injection (*p* = 0.625, Fig 1E), demonstrating that increased LH^LEPR^ neuronal activity strengthens motivation to obtain food. In contrast, we found that chemogenetic inhibition of LH^LEPR/hM4D^ neurons significantly decreased the number of food pellets earned compared to vehicle control and that PR responding for food pellets in the LH^LEPR/mCherry^ control mice did not change after CNO injection (two-way mixed-model ANOVA group × CNO interaction, *F*(1, 13) = 17.87, *p* = 0.001; Bonferroni post-test, **p = 0.0027; Fig 1F). Accordingly, further analysis showed that CNO injection significantly decreased the final ratio completed by the LH^LEPR/hM4D^ mice compared to vehicle control (Wilcoxon matched-pairs signed rank test, †*p* = 0.0156) and did not affect LH^LEPR/mCherry^ control mice (*p* = 0.1875, Fig 1G). Moreover, we observed graded behavioral responses during the PR task while chemogenetically activating or inhibiting LH^LEPR^ neurons at different incremental doses of CNO (one-way repeated-measures ANOVA: LH^LEPR/hM3D^ mice, *F*(3, 21) = 15.94, *p* < 0.0001, S2D Fig; LH^LEPR/hM4D^ mice, *F*(3, 21) = 19.72, *p* < 0.0001; S1D and S1E Fig). To assess whether nonspecific motor behavior accounted for the changes observed in the number of active lever presses during manipulations of LH^LEPR^ neurons, we analyzed the number of inactive lever presses during each behavioral test. We found that CNO injection (1 mg/kg i.p.) significantly increased inactive lever presses in LH^LEPR/hM3D^ mice (group × CNO interaction, *F*(1, 13) = 14.45, *p* = 0.0022; Bonferroni post-test, ***p = 0.0002; S1F Fig) but did not affect inactive responses in LH^LEPR/hM4D^ mice (group × CNO interaction, *F*(1, 13) = 0.5439, *p* = 0.4739; S1G Fig). The increased inactive responses in LH^LEPR/hM3D^ mice may be due to the overall elevated responding, as observed in both LH^LEPR/mCherry^ and LH^LEPR/hM4D^ mice responding at high levels during vehicle treatment under food-restricted conditions, as opposed to nonspecific motor effects (S1G Fig). Importantly, 2-h open field locomotion was not significantly affected by CNO injection across groups (one-way ANOVA, *F*(2, 20) = 2.024, *p* = 0.1583; Bonferroni’s multiple comparisons test, mCherry–hM3D: *p* = 0.6727, mCherry–hM4D: *p* = 0.6983; S1H Fig), suggesting that the changes in PR responding may not be due to generalized changes in locomotor activity. Significant increases on PR responding for food pellets were also observed in a separate cohort of mice during optogenetic activation of LH^LEPR^ neuronal activity (group × photostimulation interaction, *F*(1, 10) = 13.38, *p* = 0.0044; Bonferroni post-test, **p = 0.0097; S2A–S2D Fig). Open field locomotion was not increased during 3-min epochs of LH^LEPR^ photostimulation but was in fact slightly decreased compared to 3-min epochs without stimulation (group × photostimulation interaction, *F*(1, 10) = 6.353, *p* = 0.0304; Bonferroni post-test, *p = 0.0317; S2E Fig), thus providing further support for the idea that the increases in PR responding were not secondary to generalized increases in locomotor activity. Together, these results suggest that LH^LEPR^ neurons may regulate motivation for food.

To demonstrate CNO-mediated activation of LH^LEPR^ neurons *in vitro* and *in vivo*, we performed whole-cell recordings in brain slices as well as a quantitative analysis of FOS expression (a marker of cellular activity; S3 Fig). First, we confirmed that viral expression was restricted to LEPR-positive neurons in the LH. We generated *Lepr*^*Cre/+*^*;Rosa26*^*YFP/YFP*^ mice that express the fluorophore YFP in LEPR neurons by crossing *Lepr*^*Cre*^ mice to the Ai3 reporter line (*Rosa26*^*YFP*^; [35]). We then verified colocalization of LEPR- and YFP-positive neurons in the LH by immunostaining for phosphorylated STAT3 (pSTAT3), a marker of LEPR-induced signaling, following i.p. treatment with leptin or vehicle (S3A Fig) [13, 36, 37]. We next demonstrated that a Cre recombinase-dependent viral vector driving the expression of the fluorophore tdTomato (FLEX-tdTomato) specifically targeted LH^LEPR/YFP^ neurons (S3B Fig). Moreover, hM3D:mCherry fluorescence in LH^LEPR^ neurons does not colocalize with hypocretin (HCRT; orexin) or pro-melanin concentrating hormone (PMCH) immunofluorescence, further suggesting that virally-induced transgene expression was restricted to LH^LEPR^ neurons (S3C Fig).

To examine FOS expression, we systemically injected CNO (1 mg/kg i.p.) into *Lepr*^*Cre*^ mice injected with the Cre-dependent viral vectors encoding hM3D (left hemisphere; S3D Fig) and the fluorophore GFP as control (right hemisphere, S3D Fig). We observed similar numbers of cells transduced with each virus (120.7 ± 16.6 LH^LEPR/hM3D^; 102.3 ± 19.8 LH^LEPR/GFP^; two-tailed Student’s *t* test, *t*(4) = 0.72, *p* = 0.51; *n* = 3; S3E Fig), indicating similar levels of viral transgene expression in both hemispheres. We observed that CNO administration induced significantly more FOS-positive LH^LEPR/hM3D^ neurons compared to LH^LEPR/GFP^ (76.2 ± 6.1; LH^LEPR/hM3D/FOS+^; 9.1 ± 3.0; LH^LEPR/GFP/FOS+^; two-tailed Student’s *t* test *t*(4) = 9.91, ***p = 0.0006; *n* = 3; S3F Fig). Furthermore, we recorded action potentials from LH^LEPR/hM3D^ neurons in brain slices triggered by bath application of CNO (5 μM; *n* = 3 cells; *n* = 3 mice) in current-clamp configuration (S3G Fig). We demonstrated that CNO significantly increases the spontaneous firing rate of LH^LEPR/hM3D^ neurons (repeated-measures ANOVA with multiple comparisons, *F*(2, 4) = 16.45, *p = 0.0118; S3H Fig) and verified that the depolarizing effects of CNO are detected even after tetrodotoxin-induced blockade of action potentials (TTX; 1 μM; *n* = 3 cells; *n* = 3 mice; two-tailed Student’s *t* test, *t*(2) = 5.69, *p = 0.0295, S3I and S3J Fig). Together, these results demonstrate *in vitro* and *in vivo* specific activation of LH^LEPR/hM3D^ neurons by CNO.

### Activation of LH^LEPR^ axonal projections in the VTA promotes motivation for food

**Fig 2:**
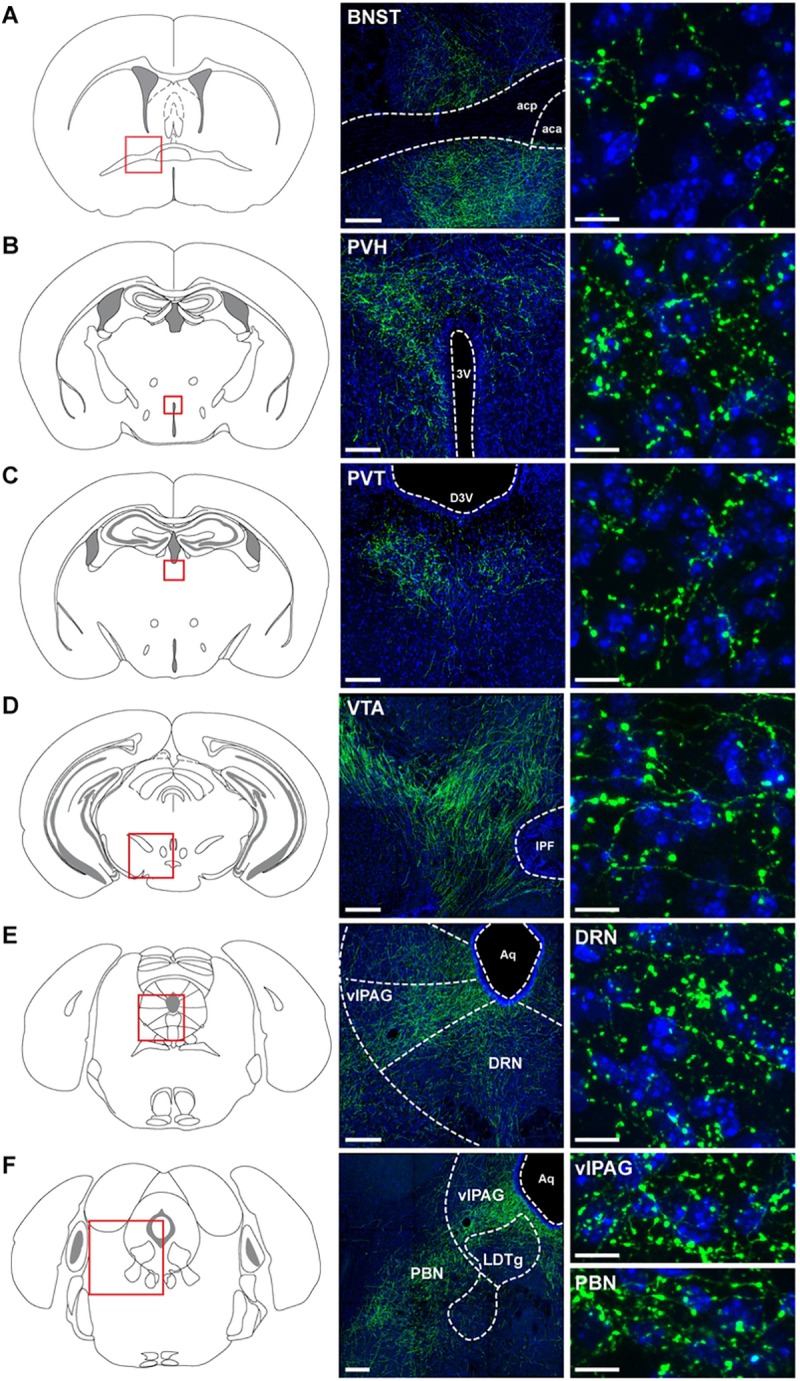
Axonal projections of LH^LEPR^ neurons. Schematic and representative images depicting LH^LEPR^ axonal projections in (**A**) bed nucleus of the stria terminalis (BNST), (**B**) paraventricular nucleus of the hypothalamus (PVH), (**C**) paraventricular nucleus of the thalamus (PVT), (**D**) ventral tegmental area (VTA), (**E**) dorsal raphe nucleus (DRN), (**F**) ventrolateral periaqueductal grey (vlPAG), and parabrachial nucleus (PBN). (A-D-E-F) Scale bars (low magnification), 200 μm; Scale bars (high magnification), 10 μm. (B and C) Scale bars (low magnification), 100 μm; Scale bars (high magnification), 10 μm. (*n* = 3 mice). Schematic images modified from Franklin KBJ & Paxinos G [40]. Abbreviations: 3rd ventricle (3V); anterior commissure, anterior part (aca); anterior commissure, posterior nerve (acp); aqueduct (Aq); dorsal 3rd ventricle (D3V); interpeduncular fossa (IPF); laterodorsal tegmental nucleus (LDTg).

**Fig 3:**
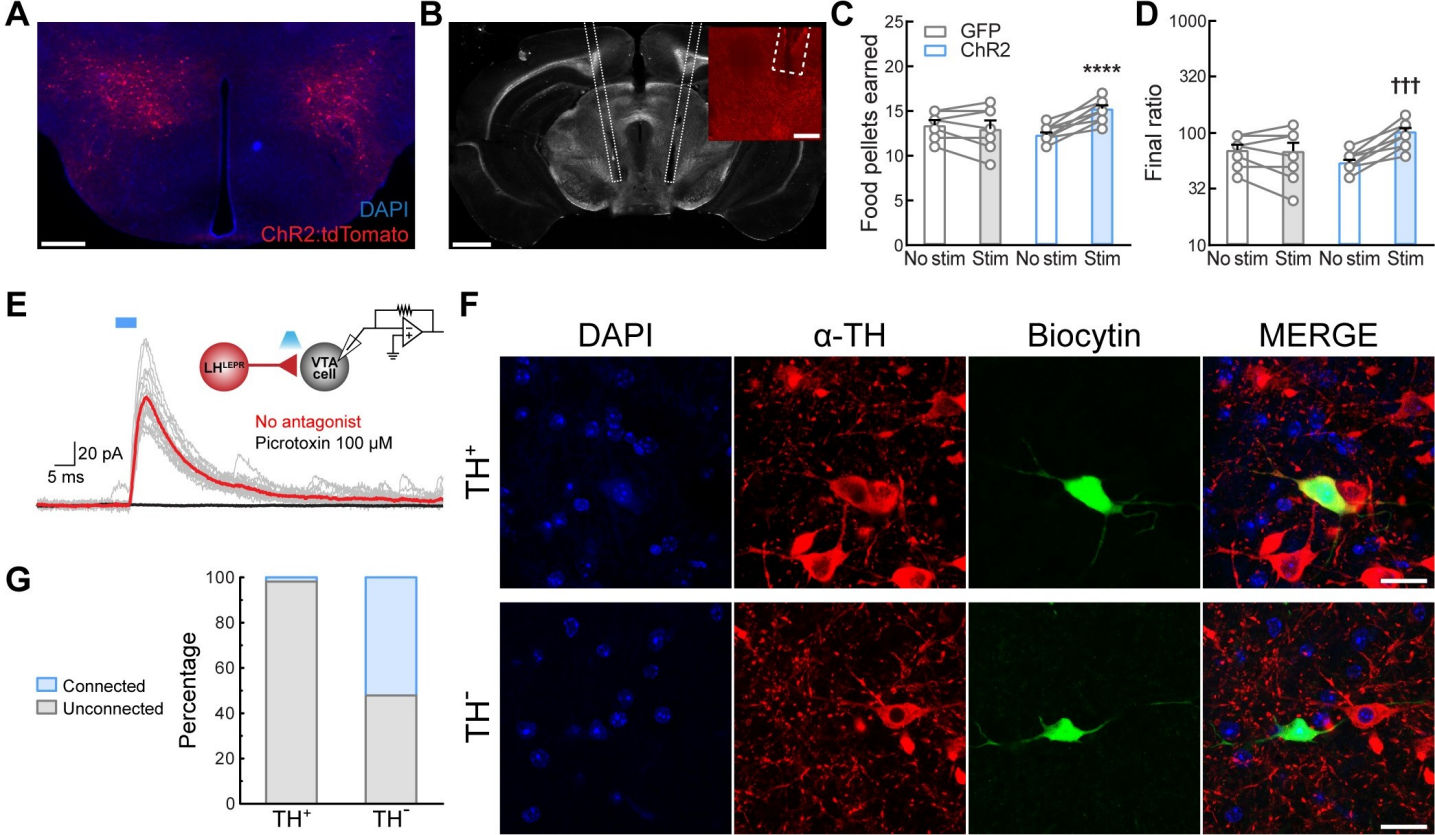
Activation of LH^LEPR^→VTA circuitry promotes motivation for food reward. Representative images showing (**A**) expression of ChR2:tdTomato in LH^LEPR^ neurons and (**B**) optical fibers implanted bilaterally above the LH^LEPR^-ChR2^+^ axonal projections in the VTA. Scale bars: (A) 500 μm, (B) 1 mm, (B, inset) 200 μm. Optogenetic activation of LH^LEPR^ axonal projections in the VTA significantly increased the number of (**C**) food pellets earned and (**D**) the final ratio completed over a two-hour session. Bars represent mean ± s.e.m.; circles indicate data from individual mice. *n* = 11 LH^LEPR/ChR2^→VTA mice and *n* = 7 LH^LEPR/GFP^→VTA control mice. Two-way ANOVA with Bonferroni post-test for pellets earned, ****p < 0.0001. Non-parametric Wilcoxon matched-pairs signed rank test for final ratio, ^†††^*p* < 0.001. (**E**) Representative traces of inhibitory postsynaptic currents (IPSCs; gray) evoked by photostimulation (5 ms blue light pulses, 0.1 Hz) of LH^LEPR^-ChR2^+^ axons in the VTA before (red trace) and after (black trace) bath application of picrotoxin (GABA_A_ receptor antagonist). Note schematic of ChR2-assisted circuit mapping (inset; upper right). (**F**) Immunohistochemical identification of VTA neurons filled with biocytin following electrophysiological recordings; representative example of a TH^+^ neuron (upper row) and TH^-^ neuron (lower row). Scale bar: 20 μm. (**G**) Summary bar graph showing the percentage of TH^+^ and TH^-^ VTA neurons that exhibited a synaptic response during photostimulation of LH^LEPR^-ChR2^+^ axons.

We next mapped the axonal projections of LH^LEPR^ neurons by injecting an adeno-associated virus into the LH of *Lepr*^*Cre*^ mice (either rAAV2/9-CAG-FLEX-eGFP-WPRE-bGH or rAAV2/9-hEf1α-DIO-synaptophysin-mCherry) [28]. Similar to previous findings [13, 38], this anterograde tracing showed dense LH^LEPR^ projections to several brain regions, including the bed nucleus of the stria terminalis (BNST), the paraventricular nucleus of the hypothalamus (PVH), the paraventricular nucleus of the thalamus (PVT), the VTA, the dorsal raphe nucleus (DRN), the ventrolateral periaqueductal grey (vlPAG), and the parabrachial nucleus (PBN) (Fig 2). Since recent studies have demonstrated that lateral hypothalamic GABAergic projections to the VTA play an important regulatory role in reward-related processing [22], we sought to determine whether LH^LEPR^ neurons promote motivation for food through connections with the VTA. To test this, we first stereotaxically injected a Cre recombinase-dependent viral vector bilaterally into the LH of *Lepr*^*cre*^ transgenic mice to target channelrhodopsin-2 (ChR2) fused to the fluorophore tdTomato (ChR2:tdTomato) [39] specifically to LH^LEPR^ neurons (Fig 3A and S4A Fig). Next, we implanted optical fibers bilaterally in the VTA to specifically activate LH^LEPR^ axonal projections (Fig 3B). Photostimulation of ChR2-expressing LH^LEPR^ axons in the VTA (LH^LEPR/ChR2^→VTA) evoked a significant increase in the number of pellets earned compared to no photostimulation and LH^LEPR/GFP^→VTA mice as control (group × photostimulation interaction, *F*(1, 16) = 30.97, *p* < 0.0001; Bonferroni post-test, ****p < 0.0001; Fig 3C). Accordingly, further analysis showed that photostimulation significantly increased the final ratio completed by LH^LEPR/ChR2^→VTA mice (Wilcoxon matched-pairs signed rank test, †††*p* = 0.001, Fig 3D) but did not affect LH^LEPR/GFP^→VTA control mice (*p* > 0.99), suggesting that LH^LEPR^ neurons may function through connections in the VTA to regulate motivation for food. Moreover, we observed graded behavioral responses during the PR task while photostimulating LH^LEPR^ axonal projections in the VTA at different frequencies (one-way repeated-measures ANOVA, *F*(2, 20) = 27.86, *p* < 0.0001; S4B Fig), indicating a frequency-dependent increase in responding. No significant changes in the number of inactive lever presses during photostimulation of the LH^LEPR^→VTA pathway were observed (group × photostimulation interaction, *F*(1, 16) = 3.287, *p* = 0.0887; S4C Fig), demonstrating that during activation of this pathway mice selectively engaged in active lever pressing to obtain food.

Thus far, our results suggest a functional LH^LEPR^→VTA pathway mediating increased motivation for food. However, we wanted to determine the nature of the synaptic interaction of these LH^LEPR^ axonal projections on neurons in the VTA. To test this, we performed channelrhodopsin-assisted circuit mapping (CRACM) in brain slices [39, 41]. We specifically targeted ChR2 to LH^LEPR^ neurons with a Cre recombinase-dependent viral vector. Next, we performed whole-cell recordings from individual neurons within the VTA (*n* = 77) under voltage-clamp configuration (Fig 3E and 3F). We observed that photostimulation of ChR2-expressing LH^LEPR^ axons evoked inhibitory postsynaptic currents (IPSCs; 123.20 ± 29.92 pA) in synaptically-connected VTA neurons (*n* = 13; 16.88% connected, Fig 3E). These IPSCs were blocked by selective antagonists of GABA_A_ receptors. This demonstrates that LH^LEPR^ neurons provide functional inhibitory synaptic inputs to neurons within the VTA. Previous studies showed that lateral hypothalamic GABAergic neurons primarily synapse onto GABAergic cells in the VTA [22], although some inputs to VTA dopaminergic (DA) neurons were also observed. Therefore, we further examined the nature of the VTA neurons targeted by the LH^LEPR^ inputs using immunohistochemical detection of tyrosine hydroxylase (TH; the rate-limiting enzyme in DA synthesis, Fig 3F) following whole-cell recordings from connected cells filled with biocytin. Consistent with previous studies [22], we found that LH^LEPR^ neurons form synapses primarily on non-dopaminergic (TH^-^; TH-negative) neurons in the VTA (VTA^TH-^: *n* = 12/23; VTA^TH+^: *n* = 1/54; Fig 3G). Therefore, these data suggest that LH^LEPR^ neurons likely regulate motivation for food by modulating the activity of VTA^TH-^ neurons.

### LH^LEPR^ neuronal activity regulates motivation for water

**Fig 4:**
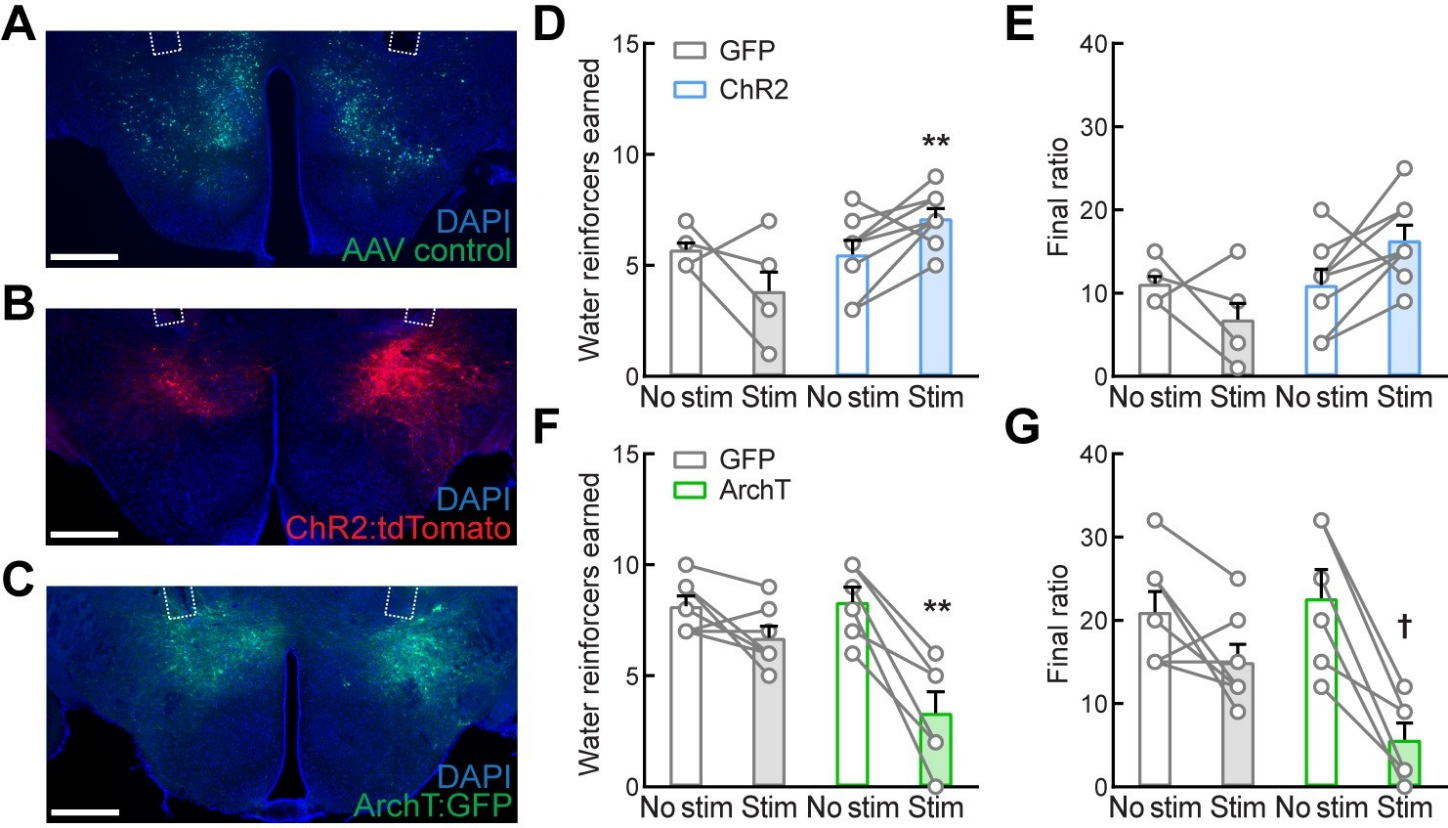
Motivation for water reward is gated by LH^LEPR^ neuronal activity. (**A−C**) Representative images depicting the expression of (**A**) GFP, (**B**) ChR2:tdTomato, and (**C**) ArchT:GFP in LH^LEPR^ neurons and optical fibers implanted bilaterally above the LH. Scale bars: 500 μm. (**D**) Photostimulation significantly increased the number of water reinforcers earned and (**E**) final ratio completed over a 2-hr session by LH^LEPR/ChR2^ mice as compared to LH^LEPR/GFP^ control mice. (**F**) Photoinhibition significantly decreased the number of water reinforcers earned and (**G**) final ratio completed by LH^LEPR/ArchT^ mice as compared to LH^LEPR/GFP^ control mice. Bars represent mean ± s.e.m.; circles indicate data from individual mice. *n* = 7 LH^LEPR/GFP^, *n* = 8 LH^LEPR/ChR2^, and *n* = 6 LH^LEPR/ArchT^ mice. Two-way ANOVA with Bonferroni post-test, **p < 0.01. Non-parametric Wilcoxon matched-pairs signed rank test for final ratio completed, ^†^*p* < 0.05.

To further investigate the functional roles of LH^LEPR^ neurons in gating motivation, we sought to determine whether these neurons promote motivation specific for food rather than other drives. To test this, we measured the effects of optogenetic manipulation of LH^LEPR^ neuronal activity on motivation to obtain water. We first targeted ChR2:tdTomato, ArchT:GFP (light-sensitive neuronal silencer), or GFP (control) specifically to LH^LEPR^ neurons. Next, we implanted optical fibers bilaterally above these neurons (Fig 4A–4C and S5A and S5B Fig). Following recovery, mice were gradually water-restricted and trained to lever press for water on a PR schedule of reinforcement. The access to water was limited to 1 min after training sessions and food was always available in their home cages. After learning the task, LH^LEPR/ChR2^ mice were returned to *ad libitum* water access in their home cages during testing, whereas LH^LEPR/ArchT^ mice remained under water restriction. One group of control LH^LEPR/GFP^ mice was trained and tested first under water restriction, and then returned to *ad libitum* water access for several days before further testing. We found that LH^LEPR/ChR2^ mice earned significantly more water reinforcers during photostimulation than LH^LEPR/GFP^ control mice (group × photostimulation interaction, *F*(1, 13) = 11.16, *p* = 0.0053; Bonferroni post-test, **p = 0.0011; Fig 4D). The final ratio completed trended towards significance for ChR2 mice (Wilcoxon matched-pairs signed rank test, *p* = 0.0859; Fig 4E). In contrast, optogenetic inhibition of LH^LEPR/ArchT^ neurons significantly decreased the number of water reinforcers earned compared to LH^LEPR/GFP^ control mice (group × photostimulation interaction, *F*(1, 11) = 10.87, *p* = 0.0071; Bonferroni post-test, **p = 0.0028; Fig 4F). The final ratio completed by the ArchT group was significantly decreased during the photoinhibition session (Wilcoxon matched-pairs signed rank test, †*p* = 0.0313; Fig 4G). Thus, these findings implicate LH^LEPR^ neurons in gating motivation to obtain water and suggest that these neurons may play a more generalized role in motivation than one directed specifically toward food. Due to this generalized role in motivation, we used *in situ* hybridization to better characterize this population of neurons. We found that approximately 68% of LH^LEPR^ neurons co-express neurotensin (*Nts*) mRNA (S6 Fig). This population was previously found to regulate mesolimbic dopamine levels [42] and thus may be primarily responsible for the effects on motivation observed in this study.

## Discussion

For decades, the LH has been depicted as a critical neuroanatomical component of a circuit mediating motivated behaviors [6]. However, the functional roles of genetically-distinct lateral hypothalamic neuronal types in orchestrating such behaviors have not been thoroughly identified. Here, we demonstrate for the first time that LH^LEPR^ neurons are key elements within the hypothalamic-ventral tegmental circuitry that gates motivation for food and water.

Our findings that cell type-specific LH^LEPR^ neuronal activation and inhibition increases and decreases lever-pressing behavior, respectively, in mice responding on a PR schedule of reinforcement for food or water directly implicates LH^LEPR^ neurons in the regulation of appetitive and motivated behaviors. Surprisingly, previous studies have shown that chemogenetic activation of lateral hypothalamic GABAergic neurons triggers food intake but does not significantly increase PR performance to obtain a palatable and caloric liquid reward [20]. Therefore, our work identifies LH^LEPR^ neurons as a distinct functional component within the LH GABAergic circuitry for motivated behavior. However, it is possible that differences in training protocols (*i*.*e*. fixed ratio *versus* progressive ratio) account for such discrepancies as a recent study has shown that chemogenetic activation of lateral hypothalamic GABAergic neurons increases overall food consumption, PR performance, and compulsive-like locomotor activity [43]. Since GABAergic neurons in the LH appear to be critical for determining the relevance of contextual stimuli [44], these discrepancies in training may be important determinants of how the LH GABAergic circuitry influences operant responding.

Interestingly, increased LH^LEPR^ neuronal activity seems to be sufficient to trigger motivation for food or water reinforcers irrespective of metabolic or hydration status as the LH^LEPR/hM3D^ and LH^LEPR/ChR2^ mice, which displayed increases in lever-pressing and the number of rewards earned, were tested under food- and water-sated conditions, whereas the LH^LEPR/hM4D^ and LH^LEPR/ArchT^ mice, which showed decreases in PR performance, were tested under food or water restriction. While it is possible that stimulating LH^LEPR^ neurons may have also increased motivation for food or water even in food- or water-restricted mice, ceiling effects could have masked such changes. This was previously observed when testing goal-directed behaviors aimed at food acquisition under fasted conditions [27], and therefore was not tested in the current study. Additionally, since we only tested the effects of activating LH^LEPR^ projections to the VTA on food motivation, further analyses are required to determine whether LH^LEPR^ neurons function through connections in the VTA or other downstream neuronal circuits to modulate motivation for water.

As predicted, our results demonstrate specific *in vivo* and *in vitro* activation of LH^LEPR/hM3D^ neurons by CNO. We observed a significant increase in neuronal activity (*i*.*e*. FOS levels) and firing rate in LH^LEPR/hM3D^ neurons after systemic or bath application of CNO, respectively. Moreover, we did not observe sedative-like behavior after CNO administration in any of the cohorts (LH^LEPR/hM3D^, LH^LEPR/hM4D^, and LH^LEPR/mCherry^ mice), as reported in a recent study [45]. At the circuit level, our LH^LEPR^ axonal projection map and those of others [13, 38] show that these neurons project to brain regions involved in feeding (BNST, PVH, and PVT) and reward and motivation (VTA and DRN), as well as other areas such as the vlPAG and PBN. Though previous studies showed dense LH^LEPR^ innervation of the VTA and suggested their potential role in reward processing and motivated behaviors [13], our work demonstrates that acute activation of this LH^LEPR^ axonal projection promotes motivation for food in mice that have been trained under a PR schedule responding for food pellets. Moreover, we show that LH^LEPR^ neurons release the neurotransmitter GABA and form functional synapses that provide inhibitory inputs to neuronal circuits within the VTA. Furthermore, our findings that LH^LEPR^ neurons primarily synapse onto non-dopaminergic neurons in the VTA (VTA^TH-^) reveal a level of functional control of the LH^LEPR^→VTA pathway and its regulation of motivation for food. Our results also extend findings from previous studies showing that lateral hypothalamic GABAergic neurons mainly form synapses with putative GABAergic cells in the VTA to increase motivated behaviors [22]. Based on what we have just described, our study identifies LH^LEPR^ neurons as a key element within the hypothalamic-ventral tegmental circuitry that gates motivation. Thus, LH^LEPR^ neuron-mediated inhibition of GABAergic cells in the VTA may likely decrease the local inhibitory control of dopaminergic neurons to increase mesolimbic dopamine release in forebrain areas involved in motivation to seek reward. LH^LEPR^ neurons may also function by releasing other neuropeptides such as neurotensin (NTS) in the VTA. Our quantified percentage of LH *Lepr*-mRNA-positive neurons that coexpress *Nts* and *Vgat* mRNA (LH^*Lepr+/Vgat+/Nts+*^, 66%) was roughly consistent with previous work showing NTS colocalization in approximately 60% of LH^LEPR^ neurons immunohistochemically in mice and with the observation that this subset of cells projects to the VTA [42]. In this context, it is also relevant that a previous study using brain slices showed that exogenous NTS enhances neuronal activity within the VTA by potentiating glutamate transmission on VTA^TH+^ cells [46]. Therefore, we cannot rule out the possibility that LH^LEPR+/VGAT+/NTS+^ neurons use this mechanism to directly modulate the activity of dopaminergic cells and influence reward processing.

Moreover, previous studies that examined the roles of LH^NTS^ neurons, a subset of which colocalizes with *Lepr*, on behavior [42, 47, 48] showed that knockout of *Lepr* in LH^NTS^ neurons led to increased bodyweight and decreased locomotion but no changes in PR responding [42, 48]. Furthermore, chemogenetic activation of LH^NTS^ neurons increased locomotor activity and decreased bodyweight but did not alter food intake [47]. A more recent study showed an increase in locomotion and decreased food intake during the dark cycle when chemogenetically activating LH^LEPR^ neurons. Interestingly, the same study also showed that activation of the broader LH^VGAT^ neuronal population decreased locomotor activity and increased food intake [49]. In contrast, ablation of LH^GABA^ neurons did not cause locomotor deficits in mice when tested in an open field assay [20]. Consistent with this previous finding, we did not observe a robust effect on total locomotion but found significant effects in lever-pressing behaviors during activation of LH^LEPR^ neurons. Together, these results highlight that there may be critical differences between gene knockout, neuronal ablation, and acute or sustained manipulations of neuronal activity, which may explain the discrepancies between our results and those from previous studies.

Accordingly, the “satiety hormone” leptin increases the activity of a subset of LH^LEPR^ [13] and LH^NTS^ [42] neurons and this depolarization likely accounts for decreased food intake following intra-LH leptin administration [13]. The deletion of *Lepr* from LH^NTS^ neurons could thus be predicted to disrupt leptin sensing and decrease the activity of this subpopulation of cells, triggering an increase in feeding. However, these knockout mice exhibited slightly increased food intake [42] and since leptin-independent events such as inputs from excitatory neurotransmitters could activate those neurons, caution should be used when interpreting these suggestive results. Moreover, some LH^LEPR^ neurons are hyperpolarized by leptin [13] suggesting the presence of diverse subpopulations of LEPR neurons in the LH. Therefore, it is possible that bulk activation of these LH^LEPR^ neurons may lead to different behavioral outputs compared to the canonical anorectic leptin-dependent effects observed in previous studies. Differences in test environments for locomotor activity (*i*.*e*. home cages versus novel test chambers) or recording schedules (*i*.*e*. light versus dark cycles) may also account for the discrepancies observed in locomotion during chemogenetic [49] or optogenetic activation of LH^LEPR^ neurons. Further work is needed to elucidate how the intersection of these factors may influence the contributions of LH^LEPR^ neurons to behavior.

The idea that different subpopulations of lateral hypothalamic GABAergic neurons encode feeding- and reward-related behaviors is supported by a recent study showing that activation of a subset of galanin-expressing neurons in the LH (LH^GABA+/GAL+^) is sufficient to drive motivated feeding behavior [43]. Interestingly, as LH^GABA+/GAL+^ neurons project to the locus coeruleus [50] in contrast with VTA-projecting LH^LEPR+/VGAT+/NTS+^ neurons, this suggests that distinct pathways likely exist for these subsets of neurons to influence motivation and reward. However, further analyses are required to determine both up- and downstream neuronal circuits involved in orchestrating these complex behaviors.

In summary, our study has identified LH^LEPR^ neurons as novel modulators within a hypothalamic-ventral tegmental circuit that gates motivation, and we provide insight into how these neurons may interact with downstream circuits to compute for appetitive and reward-related behaviors. Our findings will serve as a basis for future models of hypothalamic-ventral tegmental circuitry that regulate behaviors essential for survival. In addition, elucidating the mechanisms that regulate the compulsive nature of food seeking and intake will allow for the identification of novel therapeutics for eating disorders.

## Supporting information

S1 Fig Viral spread and effects of CNO on lever pressing and locomotion. (**A−B**) Schematic representation of (**A**) hM3D:mCherry and (**B**) hM4D:mCherry viral injections in the LH of *Lepr*^*Cre*^ mice. Schematic images modified from Franklin KBJ & Paxinos G [40]. (**C**) Schematic depicting the PR assay. (**D**) Effect of different doses of CNO on the number of food pellets earned by LH^LEPR/hM3D^ mice. (**E**) Effect of different doses of CNO on the number of food pellets earned by LH^LEPR/hM4D^ mice; One-way ANOVA with Bonferroni post-test, *p < 0.05, ***p < 0.001, ****p < 0.0001. (**F**) Inactive lever presses for LH^LEPR/hM3D^ and LH^LEPR/mCherry^ control mice during the PR task; Two-way ANOVA with Bonferroni post-test, ***p < 0.001. (**G**) Inactive lever presses for LH^LEPR/hM4D^ and LH^LEPR/mCherry^ control mice during the PR task. (**H**) Effect of 1 mg/kg CNO on 2-hr locomotion in LH^LEPR/mCherry^, LH^LEPR/hM3D^, and LH^LEPR/hM4D^ mice. Bars represent mean ± s.e.m.; circles indicate data from individual mice. *n* = 8 mice per group for hM3D and hM4D, *n* = 7 mice for mCherry.
(TIF)

S2 Fig Effects of optogenetic manipulation of LH^LEPR^ neuronal activity on PR performance. (**A−B**) Representative images depicting the expression of (**A**) GFP and (**B**) ChR2:tdTomato in LH^LEPR^ neurons and optical fibers implanted bilaterally above the LH. Scale bars: 500 μm. Sections were counterstained with DAPI. (**C**) Schematic representation of ChR2:tdTomato viral injections in the LH of *Lepr*^*Cre*^ mice. (**D**) Photostimulation of LH^LEPR^ neurons significantly increased the number of food pellets earned during the PR task. (**E**) Open field locomotion was decreased during photostimulation of LH^LEPR^ neurons. Bars represent mean ± s.e.m.; circles indicate data from individual mice. *n* = 7 LH^LEPR/GFP^ and *n* = 5 LH^LEPR/ChR2^ mice. Two-way ANOVA with Bonferroni post-test, *p < 0.05, **p < 0.01. Schematic images modified from Franklin KBJ & Paxinos G [40].
(TIF)

S3 Fig CNO-mediated activation of LH^LEPR^ neurons *in vitro* and *in vivo*. (**A**) Representative images showing phosphorylated STAT3 (pSTAT3) detected in LH^LEPR/YFP^ neurons after i.p. injection of leptin (5 mg/kg) in *Lepr*^*Cre/+*^*;Rosa26*^*YFP/YFP*^ mice compared to vehicle (saline) treatment (*n* = 3 mice). Scale bars: 200 μm. (**B**) Representative image depicting a Cre-dependent FLEX viral vector driving the expression of the fluorophore tdTomato (FLEX-tdTomato) restricted to LH^LEPR/YFP^ neurons. Scale bar: 200 μm. (**C**) hM3D:mCherry fluorescence in LH^LEPR^ neurons does not colocalize with hypocretin (HCRT; orexin) or pro-melanin concentrating hormone (PMCH) fluorescence. Section was counterstained with DAPI. Scale bars: 100 μm. (**D–F**) *In vivo* activation of LH^LEPR^ neurons by i.p. injection of CNO (1 mg/kg). (**D**) Representative image showing the expression of hM3D (left hemisphere) and the fluorophore GFP (right hemisphere) in LH^LEPR^ neurons. (**E**) Similar numbers of hM3D- and GFP-expressing LH^LEPR^ neurons were observed in each hemisphere (*n* = 3 mice). (**F**) After CNO treatment, FOS was predominantly detected in the hM3D-expressing hemisphere compared to the GFP hemisphere, Student’s two-tailed *t* test, ***p < 0.001. Scale bar: 200 μm. (**G–H**) *In vitro* activation of LH^LEPR^ neurons by bath application of CNO (5 μM) in brain slices. (**G**) Representative firing activity from an LH^LEPR^ neuron in current-clamp configuration before (baseline) and after CNO. (**H**) Of note, CNO significantly increased the firing rate of LH^LEPR/hM3D^ neurons (*n* = 3 neurons; *n* = 3 mice), One-way ANOVA with Tukey post-test, *p < 0.05. (**I–J**) Depolarizing effects of CNO were detected even after tetrodotoxin-induced blockade of action potentials (*n* = 3 cells; *n* = 3 mice), Student’s two-tailed *t* test, *p < 0.05. Abbreviations: 3rd ventricle (3V); fornix (f).
(TIF)

S4 Fig PR performance during activation of the LH^LEPR^→VTA pathway depends on photostimulus frequency. (**A**) Schematic representation of ChR2:tdTomato viral injections in the LH of *Lepr*^*Cre*^ mice. Optical fiber implants were targeted above the VTA. (**B**) Effect of different photostimulus frequencies on the number of food pellets earned during the PR task. (**C**) Inactive lever presses for LH^LEPR/ChR2^→VTA and LH^LEPR/GFP^→VTA control mice during the PR task. Bars represent mean ± s.e.m.; circles indicate data from individual mice. *n* = 11 ChR2 mice and *n* = 7 GFP mice. One-way ANOVA with Bonferroni post-test, **p < 0.01, ****p < 0.0001. Schematic images modified from Franklin KBJ & Paxinos G [40].
(TIF)

S5 Fig Viral expression of ChR2 and ArchT in LH^LEPR^ neurons. (**A−B**) Schematic representations of (**A**) ChR2:tdTomato and (**B**) ArchT:GFP viral injections in the LH of *Lepr*^*Cre*^ mice. Coronal mouse brain images modified from Franklin KBJ & Paxinos G [40].
(TIF)

S6 Fig Fluorescent *in situ* characterization of neurotransmitter and neuropeptide expression in LH^LEPR^ neurons. (**A**) *In situ* hybridization assay for *Lepr* (red), *Vgat* (white), and *Nts* (neurotensin; green) with DAPI (blue) counterstain. (**B**) Pie charts depicting proportions of neurons counted with respect to each neuronal population assayed. *Lepr* mRNA was predominantly detected in neurons that express *Vgat* (92 ± 1.3% *Lepr*^*+*^*/Vgat*^*+*^). Colocalization of *Lepr*^*+*^*/Vgat*^*+*^ with *Nts* was also observed (66 ± 5.2% *Lepr*^*+*^*/Vgat*^*+*^*/Nts*^*+*^). Cell counts were performed bilaterally on every fourth brain slice. *n* = 3 mice. Scale bar, 10 μm.
(TIF)
