# Supplementary figures and images for: Activation of a lateral hypothalamic-ventral tegmental circuit gates motivation
Source: PLoS One. 2019 Jul 10;14(7):e0219522. doi: 10.1371/journal.pone.0219522 (PMC6619795; doi:10.1371/journal.pone.0219522)

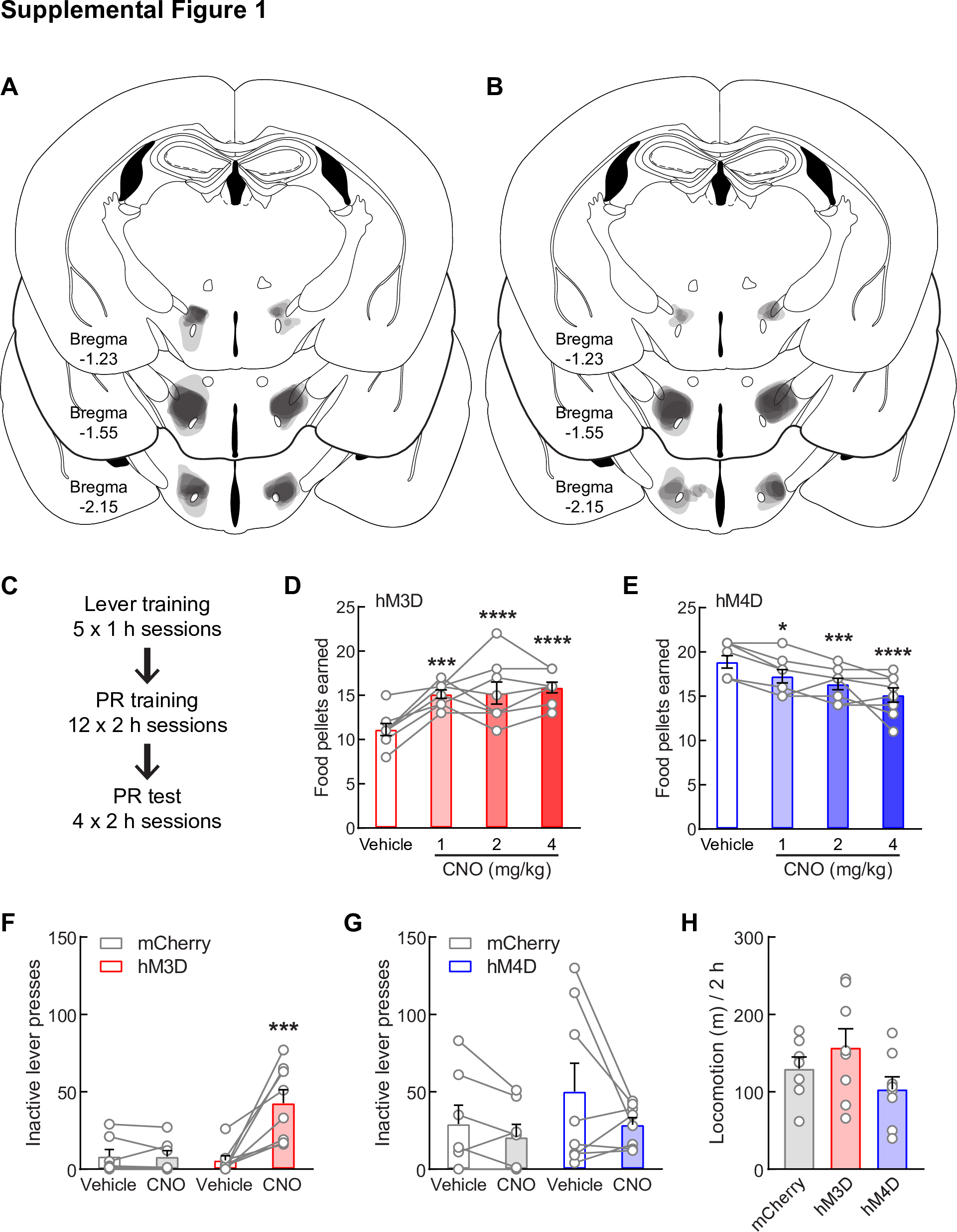

Supplement: S1 Fig — (A−B) Schematic representation of (A) hM3D:mCherry and (B) hM4D:mCherry viral injections in the LH of LeprCre mice. Schematic images modified from Franklin KBJ & Paxinos G [40]. (C) Schematic depicting the PR assay. (D) Effect of different doses of CNO on the number of food pellets earned by LHLEPR/hM3D mice. (E) Effect of different doses of CNO on the number of food pellets earned by LHLEPR/hM4D mice; One-way ANOVA with Bonferroni post-test, *p < 0.05, ***p < 0.001, ****p < 0.0001. (F) Inactive lever presses for LHLEPR/hM3D and LHLEPR/mCherry control mice during the PR task; Two-way ANOVA with Bonferroni post-test, ***p < 0.001. (G) Inactive lever presses for LHLEPR/hM4D and LHLEPR/mCherry control mice during the PR task. (H) Effect of 1 mg/kg CNO on 2-hr locomotion in LHLEPR/mCherry, LHLEPR/hM3D, and LHLEPR/hM4D mice. Bars represent mean ± s.e.m.; circles indicate data from individual mice. n = 8 mice per group for hM3D and hM4D, n = 7 mice for mCherry. (TIF) [file pone.0219522.s001.tif]

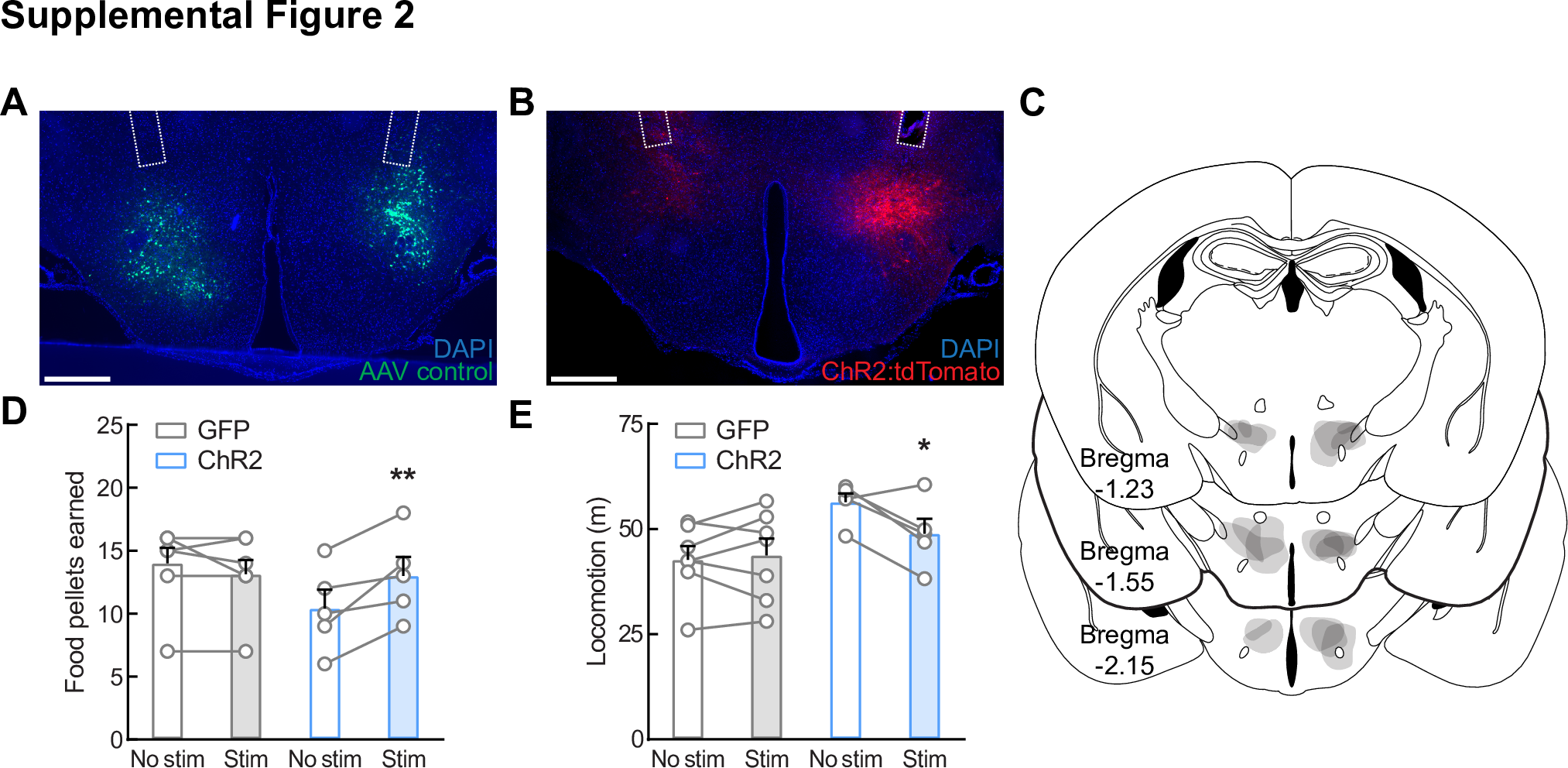

Supplement: S2 Fig — (A−B) Representative images depicting the expression of (A) GFP and (B) ChR2:tdTomato in LHLEPR neurons and optical fibers implanted bilaterally above the LH. Scale bars: 500 μm. Sections were counterstained with DAPI. (C) Schematic representation of ChR2:tdTomato viral injections in the LH of LeprCre mice. (D) Photostimulation of LHLEPR neurons significantly increased the number of food pellets earned during the PR task. (E) Open field locomotion was decreased during photostimulation of LHLEPR neurons. Bars represent mean ± s.e.m.; circles indicate data from individual mice. n = 7 LHLEPR/GFP and n = 5 LHLEPR/ChR2 mice. Two-way ANOVA with Bonferroni post-test, *p < 0.05, **p < 0.01. Schematic images modified from Franklin KBJ & Paxinos G [40]. (TIF) [file pone.0219522.s002.tif]

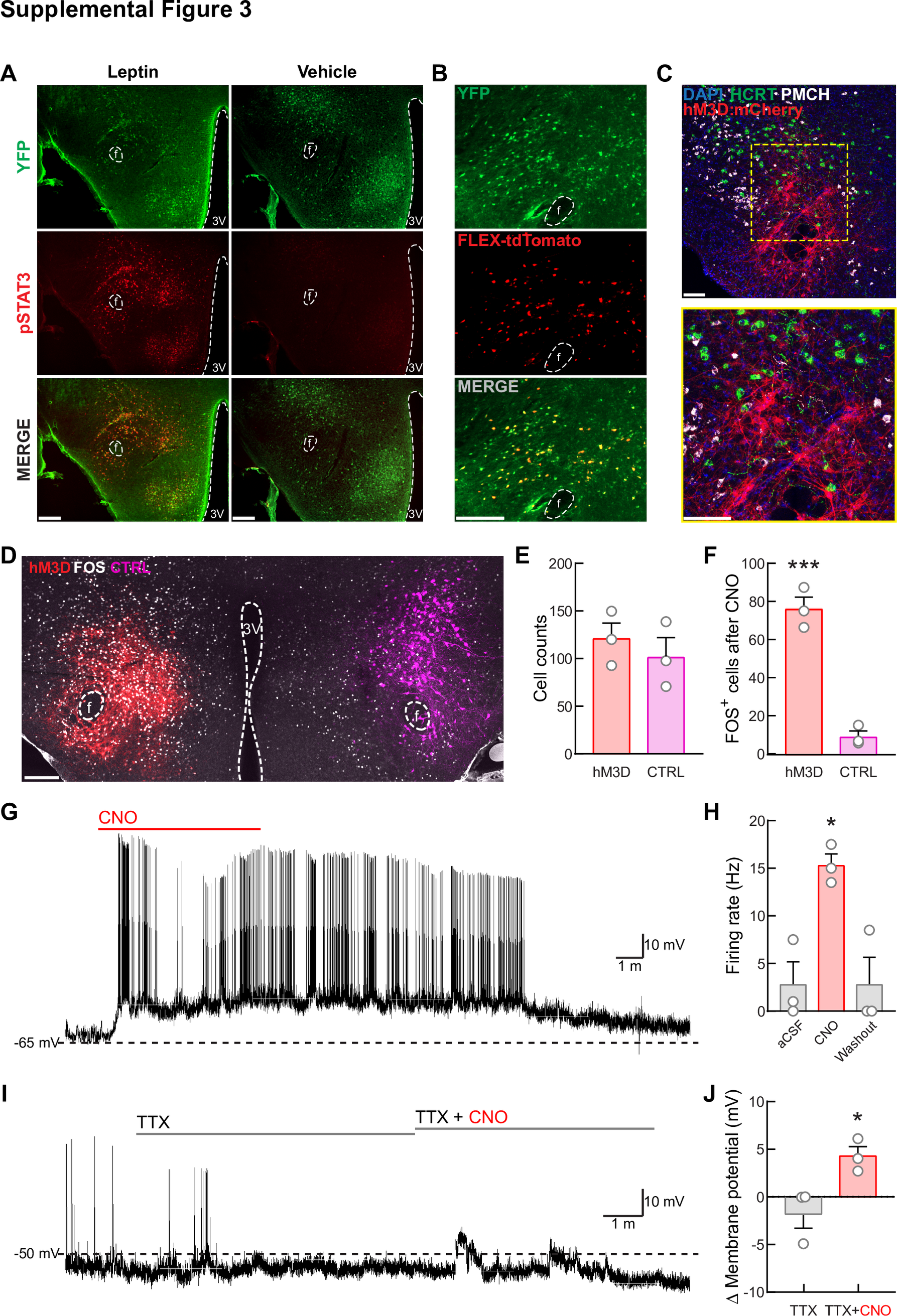

Supplement: S3 Fig — (A) Representative images showing phosphorylated STAT3 (pSTAT3) detected in LHLEPR/YFP neurons after i.p. injection of leptin (5 mg/kg) in LeprCre/+;Rosa26YFP/YFP mice compared to vehicle (saline) treatment (n = 3 mice). Scale bars: 200 μm. (B) Representative image depicting a Cre-dependent FLEX viral vector driving the expression of the fluorophore tdTomato (FLEX-tdTomato) restricted to LHLEPR/YFP neurons. Scale bar: 200 μm. (C) hM3D:mCherry fluorescence in LHLEPR neurons does not colocalize with hypocretin (HCRT; orexin) or pro-melanin concentrating hormone (PMCH) fluorescence. Section was counterstained with DAPI. Scale bars: 100 μm. (D–F) In vivo activation of LHLEPR neurons by i.p. injection of CNO (1 mg/kg). (D) Representative image showing the expression of hM3D (left hemisphere) and the fluorophore GFP (right hemisphere) in LHLEPR neurons. (E) Similar numbers of hM3D- and GFP-expressing LHLEPR neurons were observed in each hemisphere (n = 3 mice). (F) After CNO treatment, FOS was predominantly detected in the hM3D-expressing hemisphere compared to the GFP hemisphere, Student’s two-tailed t test, ***p < 0.001. Scale bar: 200 μm. (G–H) In vitro activation of LHLEPR neurons by bath application of CNO (5 μM) in brain slices. (G) Representative firing activity from an LHLEPR neuron in current-clamp configuration before (baseline) and after CNO. (H) Of note, CNO significantly increased the firing rate of LHLEPR/hM3D neurons (n = 3 neurons; n = 3 mice), One-way ANOVA with Tukey post-test, *p < 0.05. (I–J) Depolarizing effects of CNO were detected even after tetrodotoxin-induced blockade of action potentials (n = 3 cells; n = 3 mice), Student’s two-tailed t test, *p < 0.05. Abbreviations: 3rd ventricle (3V); fornix (f). (TIF) [file pone.0219522.s003.tif]

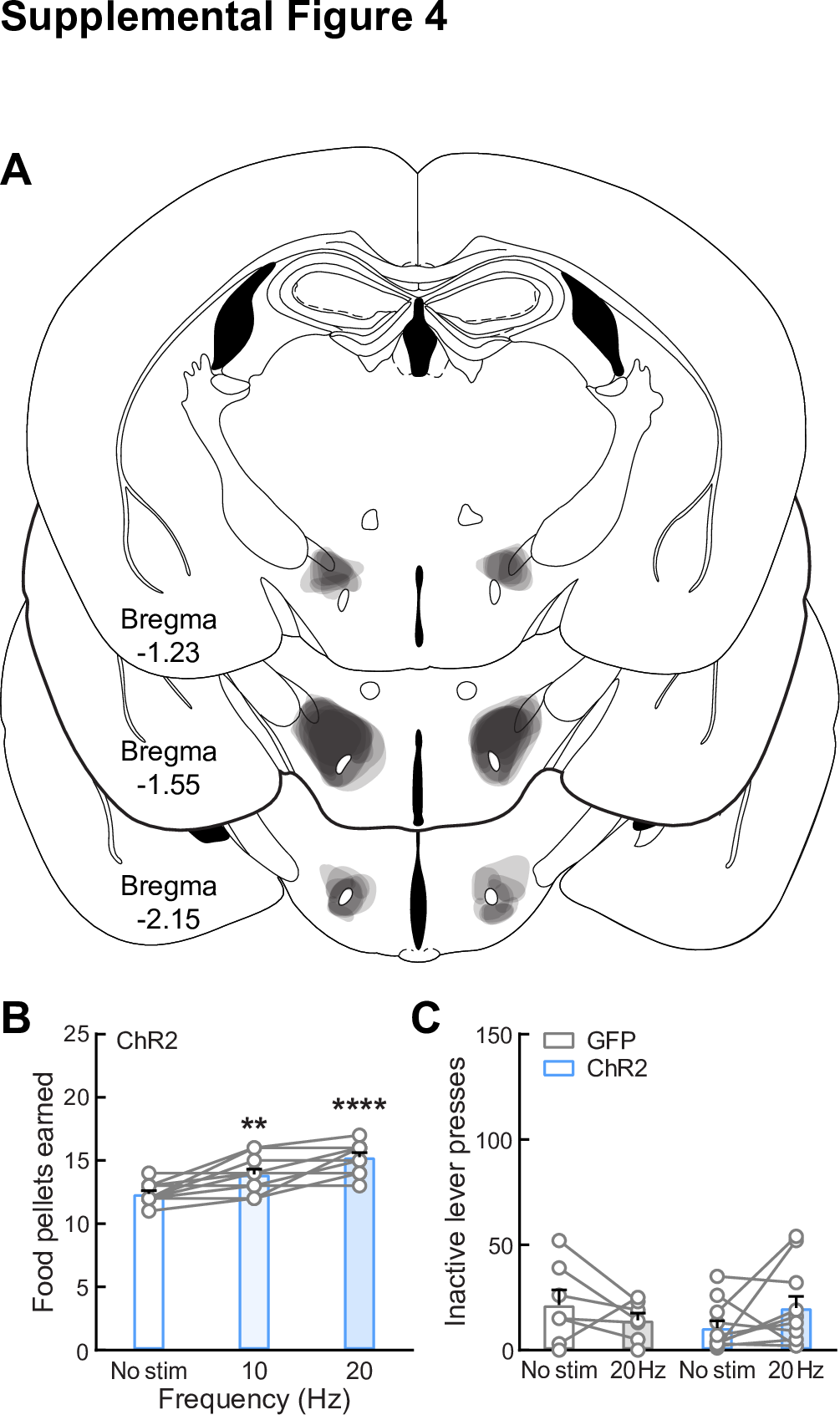

Supplement: S4 Fig — (A) Schematic representation of ChR2:tdTomato viral injections in the LH of LeprCre mice. Optical fiber implants were targeted above the VTA. (B) Effect of different photostimulus frequencies on the number of food pellets earned during the PR task. (C) Inactive lever presses for LHLEPR/ChR2→VTA and LHLEPR/GFP→VTA control mice during the PR task. Bars represent mean ± s.e.m.; circles indicate data from individual mice. n = 11 ChR2 mice and n = 7 GFP mice. One-way ANOVA with Bonferroni post-test, **p < 0.01, ****p < 0.0001. Schematic images modified from Franklin KBJ & Paxinos G [40]. (TIF) [file pone.0219522.s004.tif]

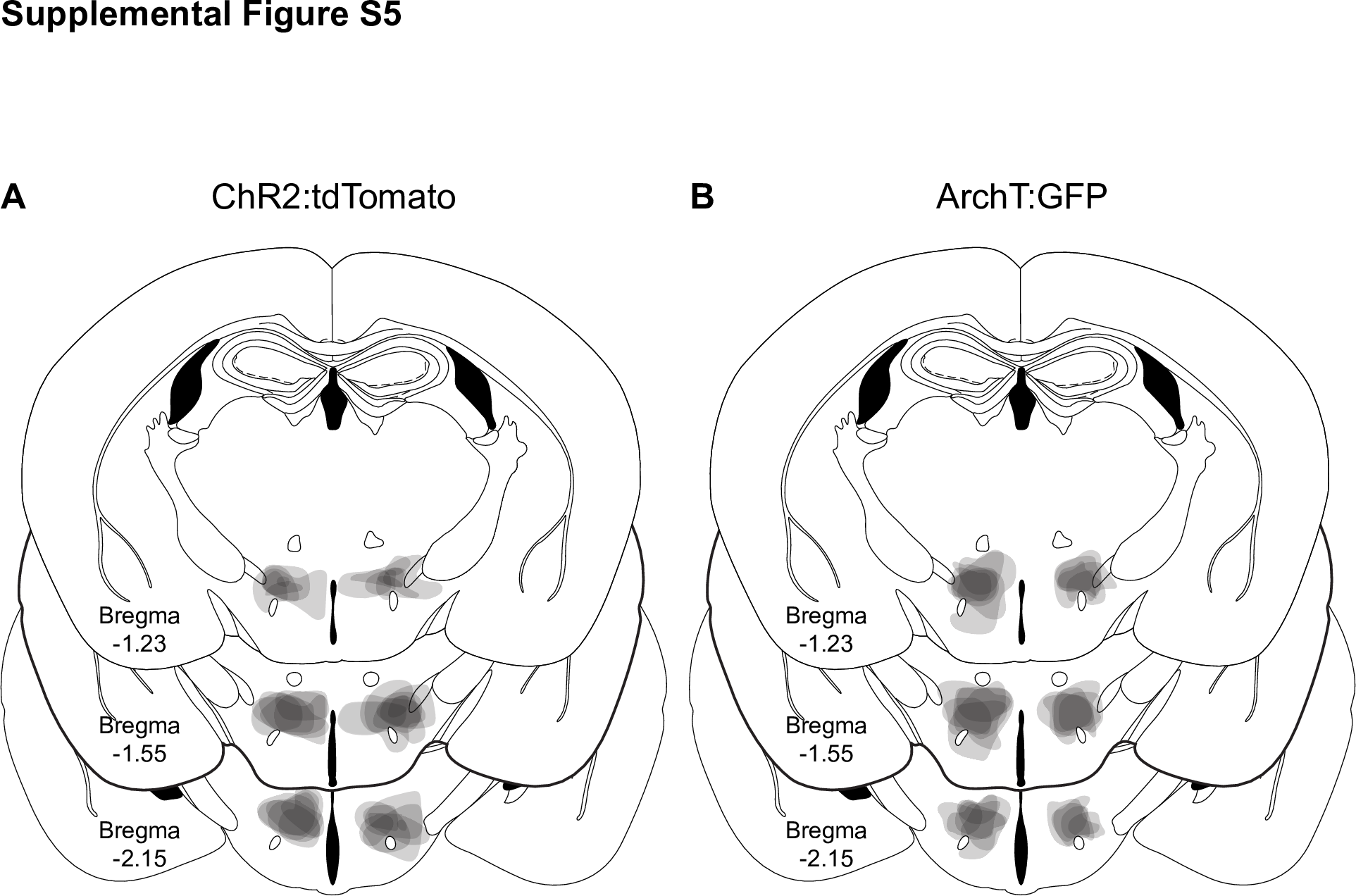

Supplement: S5 Fig — (A−B) Schematic representations of (A) ChR2:tdTomato and (B) ArchT:GFP viral injections in the LH of LeprCre mice. Coronal mouse brain images modified from Franklin KBJ & Paxinos G [40]. (TIF) [file pone.0219522.s005.tif]

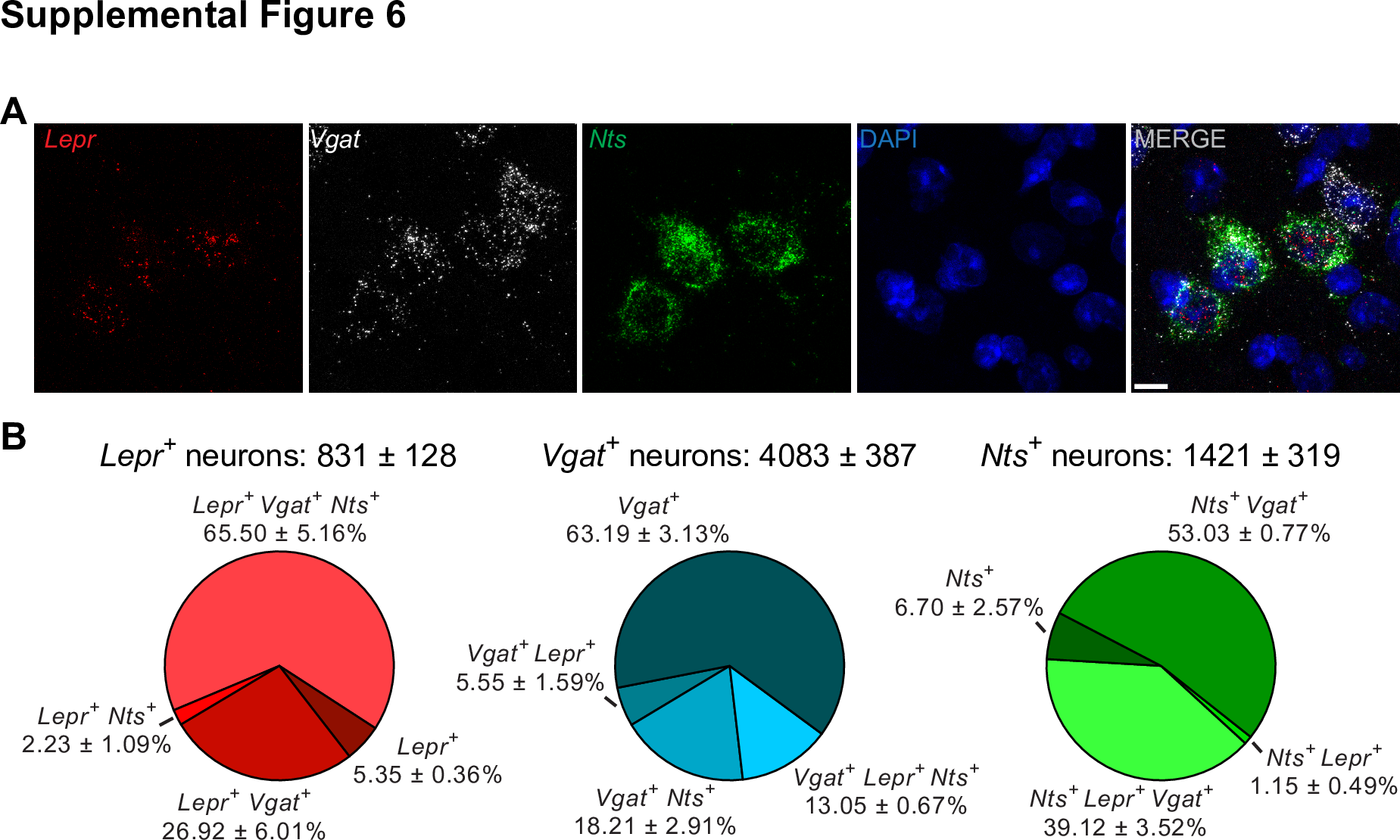

Supplement: S6 Fig — (A) In situ hybridization assay for Lepr (red), Vgat (white), and Nts (neurotensin; green) with DAPI (blue) counterstain. (B) Pie charts depicting proportions of neurons counted with respect to each neuronal population assayed. Lepr mRNA was predominantly detected in neurons that express Vgat (92 ± 1.3% Lepr+/Vgat+). Colocalization of Lepr+/Vgat+ with Nts was also observed (66 ± 5.2% Lepr+/Vgat+/Nts+). Cell counts were performed bilaterally on every fourth brain slice. n = 3 mice. Scale bar, 10 μm. (TIF) [file pone.0219522.s006.tif]
